# Identification and validation of candidate epigenetic biomarkers in lung adenocarcinoma

**DOI:** 10.1038/srep35807

**Published:** 2016-10-26

**Authors:** Iben Daugaard, Diana Dominguez, Tina E. Kjeldsen, Lasse S. Kristensen, Henrik Hager, Tomasz K. Wojdacz, Lise Lotte Hansen

**Affiliations:** 1 Department of Biomedicine, Aarhus University, Bartholins Allé 6, DK-8000 Aarhus C, Denmark; 2 Department of Pathology, Aarhus University Hospital, Nørrebrogade 45, DK-8000 Aarhus C, Denmark; 3 Aarhus Institute of Advanced Studies, Høegh-Guldbergs Gade 6B, DK-8000 Aarhus C, Denmark

## Abstract

Lung cancer is the number one cause of cancer-related deaths worldwide. DNA methylation is an epigenetic mechanism that regulates gene expression, and disease-specific methylation changes can be targeted as biomarkers. We have compared the genome-wide methylation pattern in tumor and tumor-adjacent normal lung tissue from four lung adenocarcinoma (LAC) patients using DNA methylation microarrays and identified 74 differentially methylated regions (DMRs). Eighteen DMRs were selected for validation in a cohort comprising primary tumors from 52 LAC patients and tumor-adjacent normal lung tissue from 32 patients by methylation-sensitive high resolution melting (MS-HRM) analysis. Significant increases in methylation were confirmed for 15 DMRs associated with the genes and genomic regions: *OSR1*, *SIM1*, *GHSR*, *OTX2*, *LOC648987*, *HIST1H3E*, *HIST1H3G/HIST1H2BI*, *HIST1H2AJ/HIST1H2BM, HOXD10*, *HOXD3*, *HOXB3/HOXB4*, *HOXA3*, *HOXA5*, Chr1(q21.1).A, and Chr6(p22.1). In particular the *OSR1*, *SIM1* and *HOXB3/HOXB4* regions demonstrated high potential as biomarkers in LAC. For *OSR1*, hypermethylation was detected in 47/48 LAC cases compared to 1/31 tumor-adjacent normal lung samples. Similarly, 45/49 and 36/48 LAC cases compared to 3/31 and 0/31 tumor-adjacent normal lung samples showed hypermethylation of the *SIM1* and *HOXB3/HOXB4* regions, respectively. In conclusion, this study has identified and validated 15 DMRs that can be targeted as biomarkers in LAC.

Lung cancer is the most common type of cancer and each year, the disease is responsible for approximately 1.5 million deaths worldwide^1^,^2^. There are two major types of lung cancer; small cell lung cancer (SCLC) and non-small cell lung cancer (NSCLC) accounting for 10% and 85%, of all newly diagnosed lung cancers, respectively. Lung adenocarcinoma (LAC) is the most common subtype of NSCLC, which account for approximately 40% of all lung cancers^3^. The overall 5-year survival rate for lung cancer is 15%, but the prognosis is highly dependent on the stage, at which the disease is diagnosed^4^,^5^. If the disease is localized at the time of diagnosis, the 5-year survival rate is approximately 50%, compared to approximately 25% for cases with regional disease, and less than 5% for patients that already suffer from metastatic disease^3^. Most early stage lung cancers are asymptomatic and consequently, only 15% of lung cancers are diagnosed at a local stage and more than 50% are diagnosed at an advanced stage^3^. Thus, new efficient diagnostic tools for early and accurate disease detection are needed in order to improve the poor prognosis of lung cancer.

Methylation of the carbon-5 position of cytosine residues within CpG dinucleotides is a well-established epigenetic mechanism involved in the regulation of gene expression^6^. Most CpG dinucleotides cluster in CpG rich regions in the genome, known as CpG islands (CGI), and these regions are often located within gene regulatory elements^7^. In fact, the promoter region of more than half of all protein encoding genes contain a CGI and the methylation status of this sequence is instrumental in regulating the transcriptional activity of the gene^8^. Consequently, disruption of the cell’s normal methylation pattern can have severe consequences and contribute to neoplastic transformation^7^,^8^,^9^. Genome-wide studies have shown that aberrant DNA methylation is a common feature in human cancer and hundreds of tumor suppressor genes have been shown to be subject to DNA-methylation mediated silencing^6^,^10^,^11^,^12^. Gene expression changes as a consequence of aberrant methylation have also been reported for multiple genes in lung cancer, especially hypermethylation-mediated silencing of tumor suppressor genes such as *RASSF1A*, *APC*, *RARβ*, *DAPK* and *MGMT*, and to a lesser extent, hypomethylation-mediated overexpression of proto-oncogenes such as *ELMO3*^13^,^14^,^15^,^16^,^17^.

The utility of DNA methylation biomarkers has already been established in all aspects of clinical cancer management, including risk assessment, early disease detection, prognostication and treatment personalization^18^,^19^,^20^,^21^,^22^. However, the development of biomarkers for clinical implementation is a challenging process that includes biomarker candidate discovery and evaluation of biomarker specificity and sensitivity in large-scale validation studies.

Here, we have performed a genome-wide methylation screening and identified novel methylation biomarker candidates that can potentially be used in clinical lung cancer management. We have identified cancer-specific methylation changes and performed a preliminary validation and evaluated the sensitivity and specificity of the most promising candidate biomarkers.

## Results

### Identification of differentially methylated regions (DMRs) in LAC

In order to identify novel genomic regions with disease-specific changes in methylation, we performed a genome-wide methylation screening of tumor and tumor-adjacent normal lung tissue from four LAC patients using the NimbleGen Human DNA Methylation 3 × 720 K CpG Island Plus RefSeq Promoter Array, which interrogates 15,980 CpG islands and 20,404 reference gene promoter regions^23^. After data processing, a total of 346 probes (oligonucleotides of 60 bp spotted on the array) demonstrated a significant change in methylation levels between the tumor and tumor-adjacent normal lung tissue with 288 (83.2%) and 58 (16.7%) reporting hyper- and hypomethylation in the tumor tissue, respectively. The mapping of the probes to genomic regions revealed no enrichment bias in any specific parts of the genome. Out of the 346 probes, 131 (37.9%) were located within known CGIs, 164 (47.4%) at CGI shores, 6 (1.7%) in CGI shelves and 45 (13.0%) were not associated with any known CGIs. When mapping to genes, 176 (50.9%) probes were intragenic, 128 (37.0%) were located <5 kb upstream of known genes, 3 (0.9%) were located <5 kb downstream of known genes, and 39 (11.2%) were not associated with any known gene. The 346 probes were sorted by differential methylation score, which indicates the magnitude of the detected change in methylation (See Methods section: Microarray analyses). A complete list including genomic location and position relative to known genes and CGIs is shown in Supplementary Table S1. To locate the most informative differentially methylated regions (DMRs) in LAC, we grouped the probes with a maximum inter-probe distance of 5 kb. Using this approach, we identified a total of 74 DMRs of which 63 (85.1%) showed hypermethylation and 11 (14.9%) hypomethylation in LAC. Each DMR was on average targeted by 4.1 probes (Range: 2–13 probes) and spanned 547.3 bp (Range: 126–4245 bp). Of the 74 DMRs, 65 (87.8%) and 66 (89.2%) were located in association with known genes and CGIs, respectively. Several of the identified DMRs, including *OTX2*, *OSR1* and *GHSR*, have previously been reported differentially methylated in LAC^24^,^25^. A complete list of the 74 identified DMRs, including genomic location, differential methylation scores and location relative to known genes and CGIs, is shown in Supplementary Table S2. The DMRs were sorted according to the probe with highest differential methylation score in each region and the asterisks denote the DMRs that were selected for further validation.

### Validation of candidate DNA methylation biomarkers

**Figure 1:**
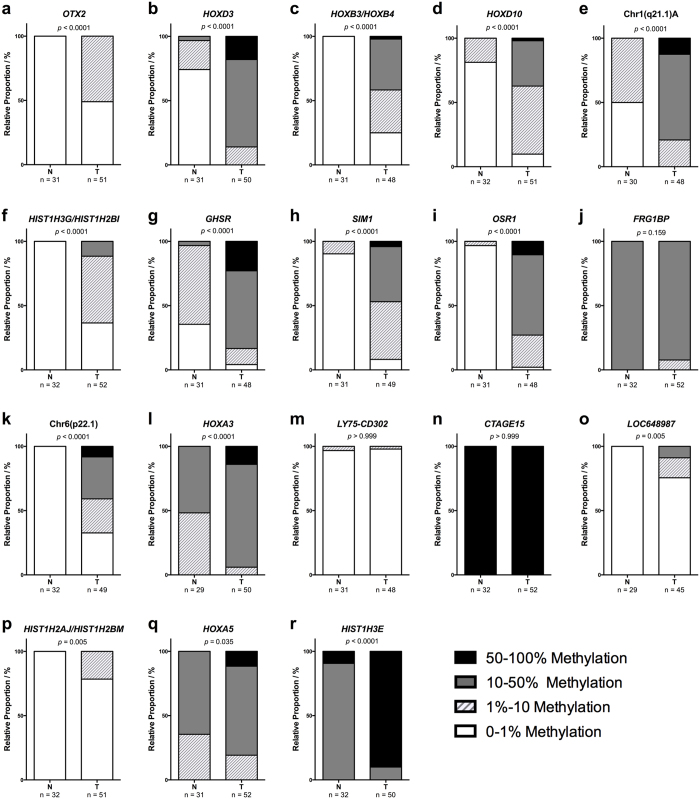
Differentially methylated regions in LAC. The methylation level of 18 DMRs was investigated in 52 LAC primary tumors and 32 tumor-adjacent normal lung samples using MS-HRM analysis. The results of the methylation assessment are shown as stacked bar percentage plots for each DMR in (**a–r)**. The relative proportion of samples in each category with 0–1% methylated templates are shown in white, 1–10% methylated templates in white with light grey stripes, 10–50% methylated templates in dark grey and 50–100% methylated templates in black. The statistical significance of the detected differences in methylation between groups was assessed using a Mann-Whitney test of ranks and two-tailed *p*-values ≤ 0.05 were considered statistically significant.

**Figure 2:**
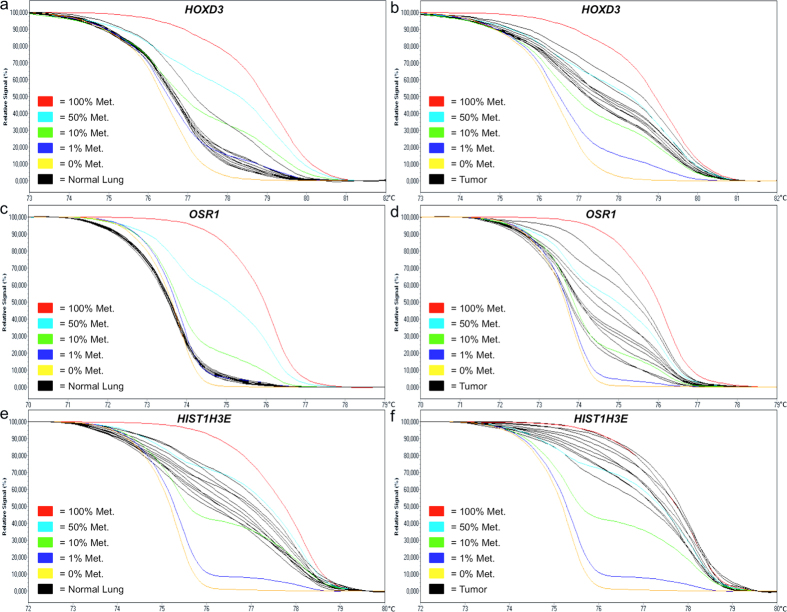
Examples of melting profiles observed for the *HOXD3*, *OSR1* and *HIST1H3E* regions. The methylation level of the 18 DMRs was determined using MS-HRM analysis. Representative normalized melting profiles for 10 tumor-adjacent normal lung samples and 10 LAC tumors are shown in black in (**a,b)** for *HOXD3*, (**c,d)** for *OSR1* and in (**e,f)** for *HIST1H3E.* The DNA methylation standards were generated as a serial dilution of fully methylated DNA into an unmethylated background. The 100% methylated standard is shown in red, 50% methylated standard in light blue, 10% methylated standard in green, 1% methylated standard in dark blue and the 0% methylated standard in yellow.

To evaluate our findings potential for clinical application, we selected 18 DMRs based upon highest differential methylation score and the number of probes targeting the region to undergo validation in a LAC validation cohort, comprising 52 primary lung tumors, 24 paired distant metastases (20 brain and 4 adrenal gland) and 32 tumor-adjacent normal lung samples. The histological and clinical characteristics of the patients are shown in Supplementary Table S3, and the selected DMRs are indicated with an asterisk in Supplementary Table S2. The array data showed that 16 of the 18 selected DMRs were hypermethylated and two regions, *FRG1BP* and *CTAGE15*, were hypomethylated in primary tumors compared to tumor-adjacent normal lung tissue. We then assessed the methylation status of each of the 18 candidate regions in our patient samples using Methylation-Sensitive High Resolution Melting (MS-HRM) analysis. The genomic location of the MS-HRM assays is shown in Table 1 and the technical specifications for each assay in Supplementary Methods. The results of the MS-HRM-based methylation assessment are summarized in Table 2 and displayed as stacked bar percentage plots in Fig. 1. Using this approach, we were able to confirm a significant increase in methylated templates in the tumor samples for 15 of the 18 selected DMRs corresponding to the hypermethylation indicated by the array analysis. Normalized melting curves for representative tumor and normal lung samples are shown in Fig. 2 for the *HOXD3*, *OSR1* and *HIST1H3E* MS-HRM assays, where the gain in methylation is seen as a relative shift in the melting curves towards the 100% methylated standard. We were unable to confirm the array results for three DMRs, including one hypermethylated region, *LY75-CD302*, and both hypomethylated regions, *FR1GB* and *CTAGE15*, as shown in Fig. 1j,m,n. For 12 of the 15 DMRs with concordant MS-HRM and array results, the difference in methylation frequency between tumor and normal lung tissue was very pronounced as indicated by the *p*-values (*p* < 0.0001) in Fig. 1. As an example, an elevated methylation level was detected in 75% of the tumor samples and 0% of the normal lung samples for the *HOXB3/HOXB4* MS-HRM assay, as shown in Fig. 1c, and in 87.9% of the tumor samples and only 3.2% of the normal lung samples for the *OSR1* MS-HRM assay shown in Fig. 1i. A high methylation frequency was also observed in the brain and adrenal gland metastases for all 15 confirmed DMRs. For the majority of the DMRs, the detected increase in methylation was even more prominent in the metastases compared to primary tumors, but due to the considerable difference in average tumor content between the primary tumors and metastases samples listed in Supplementary Table S3, these groups are not directly comparable. In conclusion, we have identified and validated 15 DMRs that can be targeted as novel biomarkers in LAC.

### The candidate DNA methylation biomarkers are not predictive of metastases formation in LAC

**Figure 3:**
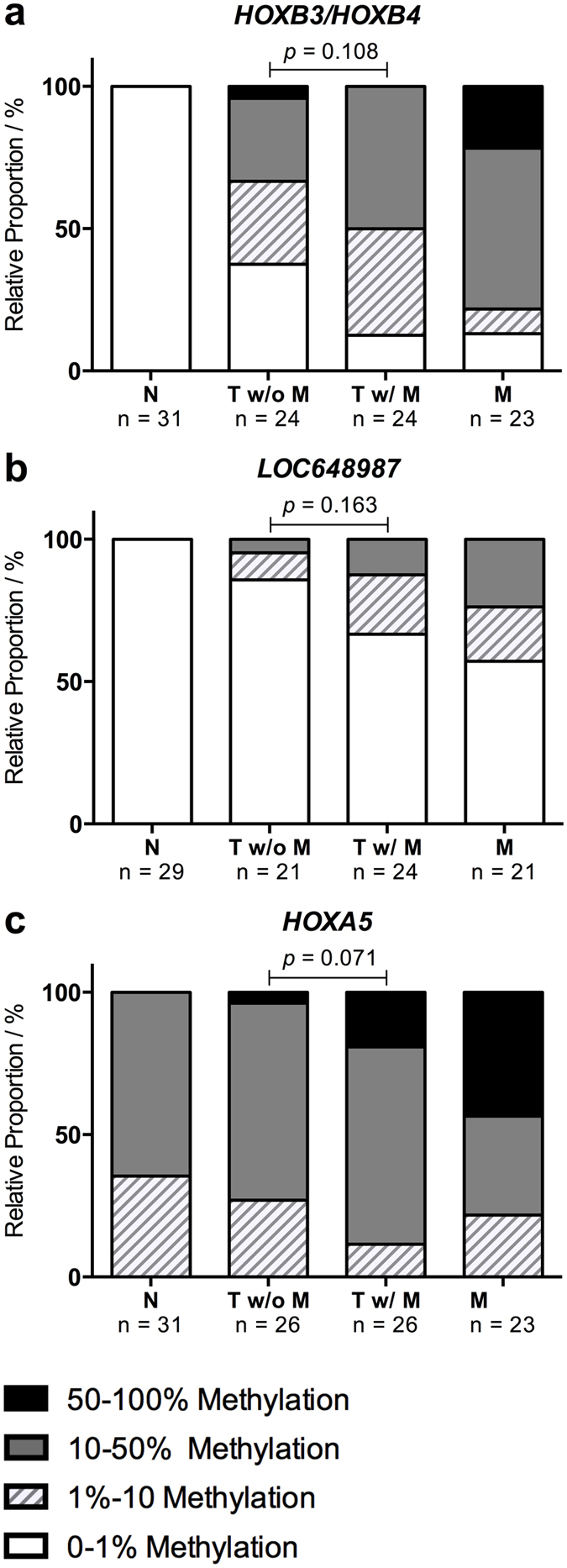
Methylation levels in metastasizing and non-metastasizing LAC tumors. The methylation levels of the 18 DMRs were compared between 26 metastasizing and 26 non-metastasizing LAC primary tumors. Three regions demonstrated a tendency towards increased methylation in the metastasizing tumors and a similar increase was observed in the 24 paired metastases (20 brain and 4 adrenal gland). The results are shown as stacked bar percentage plots in (**a**) for *HOXB3/HOXB4*, (**b**) for *LOC648987* and in (**c**) for *HOXA5.* The relative proportion of samples in each category with 0–1% methylated templates are shown in white, 1–10% methylated templates in white with light grey stripes, 10–50% methylated templates in dark grey and 50–100% methylated templates in black. The statistical significance of the detected differences in methylation between groups was assessed using a Mann-Whitney test of ranks and two-tailed *p*-values ≤ 0.05 were considered statistically significant.

The presence of metastatic disease greatly reduces the overall survival of LAC patients. To determine if hypermethylation of the 15 identified candidate biomarkers are predictive of metastases formation in LAC, we compared the methylation status of the 15 DMRs between metastases-free patients with a minimum of 5 years recurrence-free survival, and patients that suffered from distant metastatic disease at the time of diagnosis. The results are shown in Supplementary Table S4. We did not detect a significant difference in methylation frequency between the metastasizing and non-metastasizing tumors for any of the 15 DMRs. However, three of the DMRs, *HOXB3/HOXB4*, *LOC648987* and *HOXA5*, showed a trend towards an increase in methylation in the metastasizing tumors as illustrated in Fig. 3a–c. At the *HOXB3/HOXB4* region, we detected increased methylation in 15 of 24 (62.5%) non-metastasizing tumors compared to in 21 of 24 (87.5%) metastasizing tumors. Similarly, increased methylation in 3 out of 21 (14.3%) non-metastasizing tumors and in 8 out of 24 (33.3%) metastasizing tumors were detected for the *LOC648987* region, as well as in 19 out of 26 (73.1%) non-metastasizing tumors compared to 23 out of 26 (88.4%) metastasizing tumors for the *HOXA5* region. For all three DMRs, a similar increase in methylation was detected in the paired brain and adrenal gland metastases, as shown in Table 2.

### Evaluation of the clinical potential of the candidate DNA methylation biomarkers

Sensitivity and specificity are the most important parameters when describing the potential diagnostic applicability of a biomarker. In order to calculate these values, we determined an unambiguous consensus for when a sample was considered methylation positive or negative for each assay. MS-HRM is a semi-quantitative method capable of determining the relative amount of methylated alleles in a sample, and we therefore determined a specific cutoff value for each of the assays based on the relative amount of methylated alleles that is detected. For each potential cutoff value, we calculated the corresponding sensitivity and specificity, and the cutoff was then set to achieve maximal sensitivity without compromising a specificity limit of 0.8. The determined cutoff value, sensitivity and specificity for each candidate biomarker are shown in Table 3. For 9/15 assays, the cutoff was set at 1% methylation and all samples containing more than 1% methylated templates were therefore considered positive. Similarly, the cutoff was set at >10% methylation for the *HOXD3*, Chr1(q21.1).A and *GHSR* assays and at >50% methylation for the *HOXA3*, *HOXA5* and *HIST1H3E* assays. Using this approach, we achieved a specificity of ≥0.90 for all assays, except *HOXD10*, and 9/15 assays reached a specificity of 1.00, which translates into a false positive rate of 0%. The sensitivity ranged from 0.12 to 0.98 with 8/15 assays demonstrating a sensitivity of ≥0.75.

While it is possible to successfully employ cutoff values when using MS-HRM analysis, it is still preferential to use biomarkers that do not show any methylation in the corresponding normal tissue, as this allows for more accurate and stringent analyses. To identify the most promising candidate biomarkers, we therefore applied a lower sensitivity limit of 0.75 and a lower specificity limit of 0.90 to the assays with a cutoff of >1% methylation. Three candidate biomarkers fulfilled these criteria, *OSR1*, *SIM1* and *HOXB3/HOXB4*, which are indicated in bold print in Table 3. The *OSR1* assay demonstrated a sensitivity of 0.98 and a specificity of 0.97 and correctly identified 97% of all tested samples. The *SIM1* assay correctly identified 91% of the samples and showed a sensitivity of 0.92 and a specificity of 0.90 and similarly, the *HOXB3/HOXB4* assay provided a sensitivity of 0.75 and a specificity of 1.00 and therefore correctly identified 85% of the tested samples. The *OSR1*, *SIM1*, *HOXB3/HOXB4* DMRs therefore show high clinical potential as biomarkers in LAC.

## Discussion

Lung cancer has the highest mortality rates among cancers, but the prognosis for the individual patient varies considerably depending on the stage at which the disease is diagnosed^3^. Efficient diagnostic tools that allow early and accurate disease detection are therefore of critical importance in clinical lung cancer management. Compelling evidence supporting the utility of methylation biomarkers in various aspects of cancer management, such as risk assessment, disease detection and personalization of treatment, has accumulated during the last decades^18^. In this study, we aimed to identify and validate novel DMRs in LAC that can be targeted as biomarkers. Using a microarray-based genome-wide methylation screening approach, we identified 74 genomic regions that demonstrated differential methylation in tumor and tumor-adjacent normal lung tissue. Eighteen DMRs were selected for validation by MS-HRM analysis and we were able to confirm differential methylation in 15/18 DMRs. This yields a true positive rate of 83.3%, which indicates that the obtained microarray data is of high quality, but also emphasizes the importance of a thorough validation process when performing a microarray-based genome-wide methylation screening study. The validity of the data is furthermore supported by the fact that we were able to confirm differential methylation for the *HIST1H3E* region, which is number 61 out of the 74 identified DMRs when sorted by highest differential methylation score (See Supplementary Table S2). The *HIST1H3E* region was only targeted by 3 probes and only showed a differential methylation score of 2.961 and we still confirmed a significant increase in methylation (*p* < 0.0001) in the tumor samples as illustrated in Figs 1r and 2e,f. Moreover, Rauch *et al*. recently published a similar methylation screening study using 8 LAC patient samples and there are several overlaps in the identified DMRs, which serves to confirm the validity of both studies^25^.

Most of the DMRs identified in our study showed hypermethylation in LAC. In fact, 85.1% of all of the 74 identified DMRs and all 15 confirmed DMRs were hypermethylated. This overrepresentation of hypermethylated DMRs can be explained by the fact that the microarray used in our study is designed to specifically target CpG islands and reference gene promoter regions, which are known to frequently undergo *de novo* DNA methylation during tumorigenesis^6^,^10^.

In order for a biomarker to be clinically relevant, it needs to be capable of distinguishing cancerous from healthy tissue with high sensitivity and specificity, as well as deliver unambiguous results. MS-HRM analysis allows implementation of assay-specific cutoff values, which can be useful when investigating methylation changes in regions with frequent low-level methylation in the surrounding non-cancerous tissue. However, the use of assay-specific cutoff values is challenging for clinical purposes, as a tumor-related increase in methylation can be easily masked by the normal methylation level in contaminating normal cells, which are inevitably present in surgical resections and biopsies. The tumor cell content in clinical specimens vary extensively between samples and the biomarker assessment assays therefore require a high dynamic range in order to successfully test samples with both high and low tumor content and this is difficult to achieve when introducing higher cutoff values. While this can be overcome through macro- or microdissection of each specimen prior to biomarker assessment, it greatly reduces the time-efficiency and increases the cost of the individual experiment and thus limits the clinical potential of a candidate biomarker. It is therefore highly preferential to target regions that do not show methylation in normal tissue, as any increase in methylation, regardless of the magnitude, can be attributed to the presence of cancerous cells regardless of the tumor content in the clinical specimen.

We have evaluated the sensitivity and specificity for the MS-HRM assays targeting the 15 confirmed DMRs and identified the *OSR1*, *SIM1* and *HOXB3/HOXB4* regions, which all showed minor to no methylation in the surrounding normal tissue, as the most promising biomarkers in LAC. The *OSR1* region demonstrated a remarkably high sensitivity and specificity, as we detected hypermethylation in 97.9% of the LAC tumors (n = 48) and only in 3.2% of the tumor-adjacent normal lung samples (n = 31). The odd-skipped related 1 (*OSR1*) gene encodes a zinc-finger transcription factor that was recently shown to function as a tumor suppressor in gastric cancer by activating *TP53* transcription^26^,^27^. Furthermore, *OSR1* was shown to be silenced by promoter hypermethylation in 51.8% (n = 164) of gastric cancer patients and was identified as an independent predictor of poor survival^27^. Rauch *et al*. also reported *OSR1* hypermethylation in 100% of the LACs (n = 8) tested in their study, which underlines the potential of the region as a diagnostic biomarker in LAC^25^. The single-minded homolog 1 (*SIM1*) region also showed high potential as a biomarker in LAC. *SIM1* is frequently methylated in astrocytoma and breast cancer, but this study is the first to describe hypermethylation in lung cancer^28^,^29^,^30^. A substantial subset of the 74 identified DMRs, including 5/15 of the validated DMRs, *HOXD3*, *HOXB3/HOXB4*, *HOXD10*, *HOXA3* and *HOXA5*, were associated with homeobox genes. Hypermethylation of homeobox genes is a common observation in genome-wide methylation screening studies and have been reported in several cancers, including lung cancer^25^,^31^,^32^,^33^. While the homeobox genes lack tumor subtype specificity, they may still be useful in combination with other diagnostic biomarkers in LAC. The *HOXB3/HOXB4* region showed a tendency towards increased methylation in metastasizing compared to non-metastasizing tumors. We were unable to identify any DMRs that were significantly associated with metastases formation in LAC when studying primary tumors from patients with and without distant metastases. We did detect higher methylation levels in the metastases compared to the primary tumors for the majority of the DMRs, but these groups were not directly comparable due to the substantial difference in average tumor content. However, these results shows that the hypermethylation observed in the primary tumors is maintained during the metastatic process, and suggests that it may play an important role in LAC development and progression.

This study was performed using tumor-adjacent normal lung tissue as a control due to the limited availability of lung tissue from healthy individuals. We were able to validate differential methylation in 15 DMRs, but it is possible that the low-level methylation that is observed in a small subset of the normal samples for several of the regions, e.g. *HOXD10*, *HOXD3* and *SIM1*, is a result of the use of tissue, which have been exposed to the same environmental factors as the tumor tissue. It would therefore be highly relevant to investigate if the low-level methylation observed in these regions is present in normal lung tissue from healthy individuals as well. If this is not the case, then several of the DMRs that were excluded as a result of too low specificity, in particular *HOXD3* and *HOXD10*, will hold a high clinical potential as well.

The Chr6(p22.1), *HIST1H3G/HIST1H2BI* and *OTX2* regions all demonstrated a specificity of 1.0, but they were excluded due to their lower sensitivity of 0.67, 0.63 and 0.51, respectively. It would therefore be interesting to investigate the clinical potential of these regions in combination with the three most promising regions, *OSR1*, *SIM1* and *HOXB3/HOXB4*, in a larger LAC cohort.

This study has focused on the discovery of differentially methylated regions between primary tumor and tumor-adjacent normal lung tissue and the identified candidate biomarkers can therefore potentially be applied diagnostically to separate malignant tumors from benign conditions of the lung where biopsy is indicated. Similarly, all primary tumors used in this study were adenocarcinomas and it would therefore be interesting to determine if the candidate biomarkers are specific for this subtype of lung cancer, as any candidate biomarkers with such specificity may be useful diagnostic tools for tumor sub classification. Furthermore, it would be highly relevant to investigate if the candidate biomarkers can be detected in non-invasive patient samples, such as blood or expectorates, as this would allow them to be used in screening programs of high-risk individuals, such as patients suffering from chronic obstructive pulmonary disease (COPD) and thus enable early disease detection.

In conclusion, this study has identified 74 DMRs in LAC through a genome-wide methylation screening and confirmed significant changes in methylation for 15 selected regions in a LAC patient cohort using MS-HRM analysis. These 15 DMRs can be targeted as novel diagnostic biomarkers in LAC.

## Methods

### Patient samples

The regional ethical committee (De Videnskabsetiske Komitéer Region Midtjylland, Permission No.: 1-10-72-20-14) approved this study and all experiments were performed in accordance with the approved guidelines and regulations. Formalin-fixed, paraffin embedded (FFPE) blocks of surgical resections from lung adenocarcinoma (LAC) patients were selected from the archives at the Institute of Pathology, Aarhus University Hospital. Primary lung tumor and tumor-adjacent normal lung tissue from four LAC patients were used for the microarray analysis. For validation, 52 LAC patients were selected. These patients were divided into two cohorts matched on gender, age, smoking status, histology, T-stage and proportion of tumor cells. The first cohort comprises FFPE primary tumor and paired metastatic tissue (20 brain and 4 adrenal gland) from 26 patients that suffered from distant metastatic disease at the time of diagnosis. The second cohort comprises FFPE primary tumor tissue from 26 distant metastases-free patients with a minimum of 5 years recurrence-free survival following surgical resection. The histological and clinical characteristics for the 52 patients are shown in Supplementary Table S3^17^. FFPE tumor-adjacent normal lung tissue was selected by an experienced pathologist from 32 LAC patients and used as a control cohort. Peripheral blood samples obtained from healthy medical students of both sexes were used to generate unmethylated control DNA. Written informed consent was obtained from the subjects.

### DNA extraction and Sodium Bisulfite treatment

For each FFPE sample, DNA was extracted from 5 × 10 μm sections using the QIAamp DNA FFPE Tissue Kit (Qiagen, Hilden, Germany) according to the manufacturer’s protocol. DNA was extracted from the peripheral blood (PB) samples using a modified salt precipitation protocol. In brief, 10 ml blood was incubated for 30 min at 4 °C with 40 ml Triton lysis buffer (1% Triton X-100, 10 mM Tris, 0.32 M sucrose, 5 mM MgCl_2_) and spun for 30 min at 3–4000 rpm (4 °C). The supernatant was then removed and the nuclei were washed using 0.9% NaCl. After a 10 min spin 2300 rpm, the remaining supernatant was discarded and the nuclei were lysed using 3 ml nuclei lysis buffer (24 mM EDTA, 75 mM NaCl), 230 μl 10% SDS and 25 μl pronase (20 mg/ml) and left shaking at room temperature over night. For each 3 ml nuclei lysis buffer, 1 ml saturated NaCl (6 M) was added and the mix was vigorously shaken for 15 sec. The supernatant was collected after a 15 min spin at 3000 rpm (4 °C) and transferred to a new tube. After an additional 15 min spin at 3000 rpm (4 °C), isopropanol was added (1:1) to the supernatant and gently shaken until the DNA precipitated. The precipitated DNA was then collected mechanically using a blunt end glass rod and transferred to a tube containing 400 μl double-distilled H2O. DNA concentrations were measured using a NanoDrop 1000 spectrophotometer (Thermo Scientific, Waltham, MA, USA). For MS-HRM analysis, 500 ng genomic DNA from each sample was subjected to sodium bisulfite treatment using the EZ-96 DNA Methylation-Gold™ kit (Zymo Research, Irvine, USA) according to the manufacturer´s instructions and eluted in a final volume of 52 μl.

### Microarray Analyses

The microarray based screening for differentially methylated regions (DMRs) was performed as previously described^34^. Briefly, DNA was extracted from primary tumor and tumor-adjacent normal lung tissue from four LAC patients. After DNA extraction, a methylated DNA immunoprecipitation (MeDIP) was performed in order to enrich the methylated fragments. A detailed description of the MeDIP protocol can be found in^34^. Two fractions from each sample (MeDIP enriched and input) were subsequently labeled with Cy5 and Cy3 and cohybridized to the NimbleGen Human DNA Methylation 3 × 720 K CpG Island Plus RefSeq Promoter Array (Roche/NimbleGen, Madison, WI, USA). The arrays were processed using NimbleScan software (Roche/NimbleGen, Madison, WI, USA) to generate log2 signal ratios for each probe. The ratios were then averaged within each group (tumor and normal lung) and subsequently processed by the NimbleScan software to generate a relative enrichment score for each group. The enrichment scores for each group were then subtracted to produce a differential methylation score indicating an enrichment or depletion of signal in the tumor group relative to the normal lung group. Hence, negative and positive differential methylation scores indicate potentially hypo- and hypermethylated loci in lung cancer, respectively. A threshold of 2 was applied to the differential methylation score. A large fraction of the probes with differential methylation scores ≥2 were located in close proximity and the probes that were located within 5000 bp of each other were therefore grouped into differentially methylated regions (DMRs) with at least two probes targeting each DMR. Eighteen DMRs were then selected for validation based on the differential methylation score and the number of probes that mapped to the region. Previously undescribed and hypermethylated regions were prioritized. All validation experiments were performed using MS-HRM.

### Methylation-Sensitive High-Resolution Melting (MS-HRM)

Validation of the 18 potential DMRs was performed by MS-HRM analysis^35^,^36^. The LightCycler® 480 platform (Roche, Mannheim, Germany) was used for PCR and HRM, and each reaction comprised 1× MeltDoctor^TM^ HRM Master Mix (Life Technologies, Carlsbad, CA, USA), 3 mM MgCl_2_, 500 nM of each primer and 10 ng of bisulfite modified DNA in a final volume of 10 μl. All primers were designed to amplify both methylated and unmethylated DNA as described by Wojdacz *et al*.^37^. The methylation status of each DMR was determined by comparing the melting profiles of each sample with a standard dilution series of fully methylated DNA (Universal Methylated Human DNA Standard, Zymo Research, Irvine, CA USA) into unmethylated DNA, which was generated by subjecting DNA extracted from PB to whole genome amplification (WGA) using the Illustra GenomiPhi V2 DNA Amplification Kit (GE Healthcare Life Sciences, Piscataway, NJ, USA) according to the manufacturer’s instructions. All analyses were performed in duplicates. The technical specifications for each of the 18 assays, including the genomic location of the used primers, PCR cycling and HRM protocol, as well as melting profiles of the standards are included as Supplementary Information.

### Statistical analyses and calculation of sensitivity and specificity

Statistical analyses were done using GraphPad Prism version 6 software (GraphPad Software, La Jolla, CA, USA). A Mann-Whitney Test of Ranks was used to assess the statistical significance for each DMR. To perform this test, all samples were ranked based on the determined level of methylation for each DMR; 0–1% methylation was ranked 1, 1–10% methylation was ranked 2, 10–50% methylation was ranked 3 and 50–100% methylation was ranked 4. Two-tailed *p*-values ≤ 0.05 were considered statistically significant. To evaluate the clinical potential of the candidate biomarkers, we calculated the sensitivity and specificity for each region. The sensitivity was calculated as True Positives/(True Positives + False Negatives) and the specificity was calculated as True Negatives/(True Negatives + False Positives).

## Additional Information

**How to cite this article**: Daugaard, I. *et al*. Identification and validation of candidate epigenetic biomarkers in lung adenocarcinoma. *Sci. Rep.* **6**, 35807; doi: 10.1038/srep35807 (2016).

**Publisher’s note:** Springer Nature remains neutral with regard to jurisdictional claims in published maps and institutional affiliations.

## Supplementary Material

Supplementary Information

Supplementary Table S1

Supplementary Table S2

## Figures and Tables

**Table 1:** DMRs selected for validation by MS-HRM analysis.

ID	DMR			MS-HRM assay location relative to known genes and CpG Islands (CGI)				
	No.	Diff. Methylation Score	Diff. Methylation Hypo (−)/Hyper (+)	Upstream	Intragenic	Downstream	CGI	Location (Hg38)
*OTX2*	1	9.130	+		*OTX2*		Island	chr14: 56809871–56809962
*HOXD3*	2	8.830	+	*HOXD3*			Shore	chr2: 176162676–176162747
*HOXB3/HOXB4*	3	7.625	+	*HOXB3*	*HOXB4*		Shore	chr17: 48577876–48577988
*HOXD10*	4	7.318	+		*HOXD10*		Island	chr2:176117574–176117651
Chr1(q21.1).A	5	6.765	+				Island	chr1:147080162–147080262
*HIST1H3G/HIST1H2BI*	6	6.749	+	*HIST1H3G, HIST1H2BI*			Island	chr6:26272252–26272379
*GHSR*	7	6.585	+		*GHSR*		Island	chr3:172448360–172448447
*SIM1*	8	6.191	+	*SIM1*			Island	chr6:100465031–100465125
*OSR1*	9	6.117	+		*OSR1*		Island	chr2:19357150–19357251
*FRG1BP*	10	5.838	−		*FRG1BP*		Island	chr20: 30377475–30377573
Chr6(p22.1)	13	5.653	+				Island	chr6:28207550–28207669
*HOXA3*	15	5.435	+		*HOXA3*	*HOXA4*	Island	chr7: 27124232–27124343
*LY75-CD302*	17	5.223	+		*LY75-CD302, CD302*	*LY75*	Island	chr2: 159797756–159797835
*CTAGE15*	18	5.217	−	*CTAGE15*				chr7: 143571685–143571797
*LOC648987*	19	4.790	+	*ANXA2R*	*LOC648987*		Island	chr5: 43040396–43040521
*HIST1H2AJ/HIST1H2BM*	21	4.635	+	*HIST1H2BM*	*HIST1H2AJ*		Island	chr6:27814577–27814732
*HOXA5*	22	4.522	+		*HOXA5*	*HOXA6*	Island	chr7: 27143474–27143563
*HIST1H3E*	61	2.961	+		*HIST1H3E*		Island	chr6:26225275–26225421

**Table 2:** DNA methylation frequencies in tumor-adjacent normal lung, primary lung tumors and distant metastases.

ID	Normal Lung						Lung Tumor					Metastases				*P*
	Methylation Level						Methylation Level					Methylation Level				
	0–1% n (%)	1–10% n (%)	10–50% n (%)	50–100% n (%)	*N* (%)	0–1% n (%)	1–10% n (%)	10–50% n (%)	50–100% n (%)	*N* (%)	0–1% n (%)	1–10% n (%)	10–50% n (%)	50–100% n (%)	*N*(%)	Tumor vs. Normal
*OTX2*	31 (100)	0 (0)	0 (0)	0 (0)	31 (100)	25 (49.0)	26 (51.0)	0 (0)	0 (0)	51 (100)	1 (4.4)	14 (60.8)	7 (30.4)	1 (4.4)	23 (100)	<0.0001
*HOXD3*	23 (74.2)	7 (22.6)	1 (3.2)	0 (0)	31 (100)	0 (0)	7 (14.0)	34 (68.0)	9 (18.0)	50 (100)	0 (0)	0 (0)	13 (56.5)	10 (43.5)	23 (100)	<0.0001
*HOXB3/HOXB4*	31 (100)	0 (0)	0 (0)	0 (0)	31 (100)	12 (25.0)	16 (33.3)	19 (39.6)	1 (2.1)	48 (100)	3 (13.0)	2 (8.7)	13 (56.5)	5 (21.8)	23 (100)	<0.0001
*HOXD10*	26 (81.3)	6 (18.7)	0 (0)	0 (0)	32 (100)	5 (9.8)	27 (52.9)	18 (35.3)	1 (2.0)	51 (100)	2 (8.3)	10 (41.7)	11 (45.8)	1 (4.2)	24 (100)	<0.0001
Chr1(q21.1).A	15 (50.0)	15 (50.0)	0 (0)	0 (0)	30 (100)	0 (0)	10 (20.8)	32 (66.7)	6 (12.5)	48 (100)	0 (0)	2 (8.7)	12 (52.2)	9 (39.1)	23 (100)	<0.0001
*HIST1H3G/HIST1H2BI*	32 (100)	0 (0)	0 (0)	0 (0)	32 (100)	19 (36.5)	27 (51.9)	6 (11.6)	0 (0)	52 (100)	8 (34.8)	6 (26.1)	9 (39.1)	0 (0)	23 (100)	<0.0001
*GHSR*	11 (35.5)	19 (61.3)	1 (3.2)	0 (0)	31 (100)	2 (4.2)	6 (12.5)	29 (60.4)	11 (22.9)	48 (100)	1 (4.4)	2 (8.7)	11 (47.8)	9 (39.1)	23 (100)	<0.0001
*SIM1*	28 (90.3)	3 (9.7)	0 (0)	0 (0)	31 (100)	4 (8.2)	22 (44.9)	21 (42.8)	2 (4.1)	49 (100)	1(4.6)	3 (13.6)	14 (63.6)	4 (18.2)	22 (100)	<0.0001
*OSR1*	30 (96.8)	1 (3.2)	0 (0)	0 (0)	31 (100)	1 (2.1)	12 (25.0)	30 (62.5)	5 (10.4)	48 (100)	3 (13.6)	1 (4.6)	9 (40.9)	9 (40.9)	22 (100)	<0.0001
*FRG1BP*	0 (0)	0 (0)	32 (100)	0 (0)	32 (100)	0 (0)	4 (7.7)	48 (92.3)	0 (0)	52 (100)	0 (0)	4 (17.4)	17 (73.9)	2 (8.7)	23 (100)	0.159
Chr6(p22.1)	32 (100)	0 (0)	0 (0)	0 (0)	32 (100)	16 (32.6)	13 (26.6)	16 (32.6)	4 (8.2)	49 (100)	5 (21.7)	2 (8.7)	12 (52.2)	4 (17.4)	23 (100)	<0.0001
*HOXA3*	0 (0)	14 (48.3)	15 (51.7)	0 (0)	29 (100)	0 (0)	3 (6.0)	40 (80.0)	7 (14.0)	50 (100)	0 (0)	6 (26.1)	10 (43.5)	7 (30.4)	23 (100)	<0.0001
*LY75-CD302*	30 (96.8)	1 (3.2)	0 (0)	0 (0)	31 (100)	47 (97.9)	1 (2.1)	0 (0)	0 (0)	48 (100)	22 (100)	0 (0)	0 (0)	0 (0)	22 (100)	>0.999
*CTAGE15*	0 (0)	0 (0)	0 (0)	32 (100)	32 (100)	0 (0)	0 (0)	0 (0)	52 (100)	52 (100)	0 (0)	0 (0)	1 (4.4)	22 (95.6)	23 (100)	>0.999
*LOC648987*	29 (100)	0 (0)	0 (0)	0 (0)	29 (100)	34 (75.5)	7 (15.6)	4 (8.9)	0 (0)	45 (100)	12 (57.1)	4 (19.1)	5 (23.8)	0 (0)	21 (100)	0.005
*HIST1H2AJ/HIST1H2BM*	32 (100)	0 (0)	0 (0)	0 (0)	32 (100)	40 (78.4)	11 (21.6)	0 (0)	0 (0)	51 (100)	19 (82.6)	3 (13.0)	1 (4.4)	0 (0)	23 (100)	0.005
*HOXA5*	0 (0)	11 (35.5)	20 (64.5)	0 (0)	31 (100)	0 (0)	10 (19.2)	36 (69.2)	6 (11.6)	52 (100)	0 (0)	5 (21.7)	8 (34.8)	10 (43.5)	23 (100)	0.035
*HIST1H3E*	0 (0)	0 (0)	29 (90.6)	3 (9.4)	32 (100)	0 (0)	0 (0)	5 (10.0)	45 (90.0)	50 (100)	0 (0)	0 (0)	1 (4.4)	22 (95.6)	23 (100)	<0.0001

**Table 3:** Performance of MS-HRM assays.

ID	Cutoff Value	Normal Lung			Lung Tumor			Sensitivity	Specificity
	Methylation Level	True Negative n (%)	False positive n (%)	*N* (%)	False Negative n (%)	True Positive n (%)	*N* (%)		
***OSR1***	>1%	30 (96.8)	1 (3.2)	31 (100)	1 (2.1)	47 (97.9)	48 (100)	0.98	0.97
***SIM1***	>1%	28 (90.3)	3 (9.7)	31 (100)	4 (8.2)	45 (91.8)	49 (100)	0.92	0.90
*HOXD10*	>1%	26 (81.3)	6 (18.7)	32 (100)	5 (9.8)	46 (90.2)	51 (100)	0.90	0.81
*HIST1H3E*	>50%	29 (90.6)	3 (9.4)	32 (100)	5 (10.0)	45 (90.0)	50 (100)	0.90	0.91
*HOXD3*	>10%	30 (96.8)	1 (3.2)	31 (100)	7 (14.0)	43 (86.0)	50 (100)	0.86	0.97
*GHSR*	>10%	30 (96.8)	1 (3.2)	31 (100)	8 (16.7)	40 (83.3)	48 (100)	0.83	0.97
Chr1(q21.1).A	>10%	30 (100)	0 (0)	30 (100)	10 (20.8)	38 (79.2)	48 (100)	0.79	1.00
***HOXB3/HOXB4***	>1%	31 (100)	0 (0)	31 (100)	12 (25.0)	36 (75.0)	48 (100)	0.75	1.00
Chr6(p22.1)	>1%	32 (100)	0 (0)	32 (100)	16 (32.6)	33 (67.4)	49 (100)	0.67	1.00
*HIST1H3G/HIST1H2BI*	>1%	32 (100)	0 (0)	32 (100)	19 (36.5)	33 (63.5)	52 (100)	0.63	1.00
*OTX2*	>1%	31 (100)	0 (0)	31 (100)	25 (49.0)	26 (51.0)	51 (100)	0.51	1.00
*LOC648987*	>1%	29 (100)	0 (0)	29 (100)	34 (75.5)	11 (24.5)	45 (100)	0.24	1.00
*HIST1H2AJ/HIST1H2BM*	>1%	32 (100)	0 (0)	32 (100)	40 (78.4)	11 (21.6)	51 (100)	0.22	1.00
*HOXA3*	>50%	29 (100)	0 (0)	29 (100)	43 (86.0)	7 (14.0)	50 (100)	0.14	1.00
*HOXA5*	>50%	31 (100)	0 (0)	31 (100)	46 (88.4)	6 (11.6)	52 (100)	0.12	1.00

## References

[b1] Siegel R. L., Miller K. D. & Jemal A. Cancer statistics, 2015. CA: a cancer journal for clinicians 65, 5–29, 10.3322/caac.21254 (2015). 25559415

[b2] Ferlay J. . Cancer incidence and mortality worldwide: sources, methods and major patterns in GLOBOCAN 2012. International journal of cancer 136, E359–E386, 10.1002/ijc.29210 (2015). 25220842

[b3] DeSantis C. E. . Cancer treatment and survivorship statistics, 2014. CA: a cancer journal for clinicians 64, 252–271, 10.3322/caac.21235 (2014). 24890451

[b4] Ettinger D. S. . Non-small cell lung cancer, version 2.2013. Journal of the National Comprehensive Cancer Network: JNCCN 11, 645–653, quiz 653 (2013). 10.6004/jnccn.2013.0084 23744864

[b5] Miller Y. E. Pathogenesis of lung cancer: 100 year report. American journal of respiratory cell and molecular biology 33, 216–223, 10.1165/rcmb.2005-0158OE (2005). 16107574 PMC2715312

[b6] Esteller M. Epigenetics in cancer. The New England journal of medicine 358, 1148–1159, 10.1056/NEJMra072067 (2008). 18337604

[b7] Weber M. . Distribution, silencing potential and evolutionary impact of promoter DNA methylation in the human genome. Nature genetics 39, 457–466, 10.1038/ng1990 (2007). 17334365

[b8] Illingworth R. S. & Bird A. P. CpG islands–‘a rough guide’. FEBS letters 583, 1713–1720, 10.1016/j.febslet.2009.04.012 (2009). 19376112

[b9] Jones P. A. & Baylin S. B. The fundamental role of epigenetic events in cancer. Nature reviews. Genetics 3, 415–428, 10.1038/nrg816 (2002). 12042769

[b10] Jones P. A. & Baylin S. B. The epigenomics of cancer. Cell 128, 683–692, 10.1016/j.cell.2007.01.029 (2007). 17320506 PMC3894624

[b11] Herman J. G. & Baylin S. B. Gene silencing in cancer in association with promoter hypermethylation. The New England journal of medicine 349, 2042–2054, 10.1056/NEJMra023075 (2003). 14627790

[b12] Rauch T. A. . High-resolution mapping of DNA hypermethylation and hypomethylation in lung cancer. Proceedings of the National Academy of Sciences of the United States of America 105, 252–257, 10.1073/pnas.0710735105 (2008). 18162535 PMC2224196

[b13] Dammann R. . Epigenetic inactivation of a RAS association domain family protein from the lung tumour suppressor locus 3p21.3. Nature genetics 25, 315–319, 10.1038/77083 (2000). 10888881

[b14] Dammann R., Takahashi T. & Pfeifer G. P. The CpG island of the novel tumor suppressor gene RASSF1A is intensely methylated in primary small cell lung carcinomas. Oncogene 20, 3563–3567, 10.1038/sj.onc.1204469 (2001). 11429703

[b15] Virmani A. K. . Aberrant methylation of the adenomatous polyposis coli (APC) gene promoter 1A in breast and lung carcinomas. Clinical cancer research: an official journal of the American Association for Cancer Research 7, 1998–2004 (2001). 11448917

[b16] Zochbauer-Muller S. . Aberrant promoter methylation of multiple genes in non-small cell lung cancers. Cancer research 61, 249–255 (2001). 11196170

[b17] Soes S. . Hypomethylation and increased expression of the putative oncogene ELMO3 are associated with lung cancer development and metastases formation. Oncoscience 1, 367–374, 10.18632/oncoscience.42 (2014). 25594031 PMC4278312

[b18] Baylin S. B. & Jones P. A. A decade of exploring the cancer epigenome - biological and translational implications. Nature reviews. Cancer 11, 726–734, 10.1038/nrc3130 (2011). 21941284 PMC3307543

[b19] Shi H., Wang M. X. & Caldwell C. W. CpG islands: their potential as biomarkers for cancer. Expert review of molecular diagnostics 7, 519–531, 10.1586/14737159.7.5.519 (2007). 17892361

[b20] Laird P. W. The power and the promise of DNA methylation markers. Nat Rev Cancer 3, 253–266, 10.1038/nrc1045 (2003). 12671664

[b21] Heichman K. A. & Warren J. D. DNA methylation biomarkers and their utility for solid cancer diagnostics. Clinical chemistry and laboratory medicine 50, 1707–1721, 10.1515/cclm-2011-0935 (2012). 23089699

[b22] Mikeska T., Bock C., Do H. & Dobrovic A. DNA methylation biomarkers in cancer: progress towards clinical implementation. Expert review of molecular diagnostics 12, 473–487, 10.1586/erm.12.45 (2012). 22702364

[b23] Cong L., Jia J., Qin W., Ren Y. & Sun Y. Genome-wide analysis of DNA methylation in an APP/PS1 mouse model of Alzheimer’s disease. Acta neurologica Belgica 114, 195–206, 10.1007/s13760-013-0267-6 (2014). 24347181

[b24] Moskalev E. A. . GHSR DNA hypermethylation is a common epigenetic alteration of high diagnostic value in a broad spectrum of cancers. Oncotarget 6, 4418–4427, 10.18632/oncotarget.2759 (2015). 25557172 PMC4414200

[b25] Rauch T. A. . DNA methylation biomarkers for lung cancer. Tumour biology: the journal of the International Society for Oncodevelopmental Biology and Medicine 33, 287–296, 10.1007/s13277-011-0282-2 (2012). 22143938

[b26] Katoh M. Molecular cloning and characterization of OSR1 on human chromosome 2p24. International journal of molecular medicine 10, 221–225 (2002). 12119563

[b27] Otani K. . Odd-skipped related 1 is a novel tumour suppressor gene and a potential prognostic biomarker in gastric cancer. The Journal of pathology 234, 302–315, 10.1002/path.4391 (2014). 24931004 PMC4277686

[b28] Miyamoto K. . Identification of 20 genes aberrantly methylated in human breast cancers. International journal of cancer. Journal international du cancer 116, 407–414, 10.1002/ijc.21054 (2005). 15818620

[b29] Faryna M. . Genome-wide methylation screen in low-grade breast cancer identifies novel epigenetically altered genes as potential biomarkers for tumor diagnosis. FASEB journal: official publication of the Federation of American Societies for Experimental Biology 26, 4937–4950, 10.1096/fj.12-209502 (2012). 22930747

[b30] Wu X. . CpG island hypermethylation in human astrocytomas. Cancer research 70, 2718–2727, 10.1158/0008-5472.CAN-09-3631 (2010). 20233874 PMC2848870

[b31] Rauch T. . Homeobox gene methylation in lung cancer studied by genome-wide analysis with a microarray-based methylated CpG island recovery assay. Proceedings of the National Academy of Sciences of the United States of America 104, 5527–5532, 10.1073/pnas.0701059104 (2007). 17369352 PMC1838508

[b32] Schlesinger Y. . Polycomb-mediated methylation on Lys27 of histone H3 pre-marks genes for de novo methylation in cancer. Nature genetics 39, 232–236, 10.1038/ng1950 (2007). 17200670

[b33] Tommasi S., Karm D. L., Wu X., Yen Y. & Pfeifer G. P. Methylation of homeobox genes is a frequent and early epigenetic event in breast cancer. Breast cancer research: BCR 11, R14, 10.1186/bcr2233 (2009). 19250546 PMC2687719

[b34] Wojdacz T. K. . Identification and characterization of locus specific methylation patterns within novel loci undergoing hypermethylation during breast cancer pathogenesis. Breast cancer research: BCR 16, R17, 10.1186/bcr3612 (2014). 24490656 PMC3978461

[b35] Wojdacz T. K., Dobrovic A. & Hansen L. L. Methylation-sensitive high-resolution melting. Nature protocols 3, 1903–1908, 10.1038/nprot.2008.191 (2008). 19180074

[b36] Wojdacz T. K. & Dobrovic A. Methylation-sensitive high resolution melting (MS-HRM): a new approach for sensitive and high-throughput assessment of methylation. Nucleic acids research 35, e41, 10.1093/nar/gkm013 (2007). 17289753 PMC1874596

[b37] Wojdacz T. K., Hansen L. L. & Dobrovic A. A new approach to primer design for the control of PCR bias in methylation studies. BMC Res Notes 1, 54, 10.1186/1756-0500-1-541756-0500-1-54[pii] (2008). 18710507 PMC2525644

