# Supplementary material for: Identification and validation of candidate epigenetic biomarkers in lung adenocarcinoma
Source: Sci Rep. 2016 Oct 26;6:35807. doi: 10.1038/srep35807 (PMC5080630; doi:10.1038/srep35807)

**SUPPLEMENTARY INFORMATION**

**Identification and validation of candidate epigenetic biomarkers in lung adenocarcinoma**

**Iben Daugaard^1*^, Diana D Rodriguez^1^, Tina E Kjeldsen^1^, Lasse S Kristensen^1^, Henrik Hager^2^, Tomasz K Wojdacz^1, 3^, Lise Lotte Hansen^1^**

^1^Department of Biomedicine, Aarhus University, Bartholins Allé 6, DK-8000 Aarhus C, Denmark

^2^Department of Pathology, Aarhus University Hospital, Nørrebrogade 45, DK-8000 Aarhus C, Denmark

^3^Aarhus Institute of Advanced Studies, Aarhus University, Høegh-Guldbergs Gade 6B, DK-8000 Aarhus C, Denmark

*Corresponding author: Iben Daugaard. Email: iben@biomed.au.dk. Telephone: 0045 87167776. Current address: Institute of Biomedicine, Aarhus University, Bartholins Allé 6, Building 1242-224, DK-8000 Aarhus C, Denmark.

**Supplementary Table S3: Clinical characteristics for the patient cohorts**

| **Clinical Characteristics** | **LAC**  **w/o metastases** | **LAC**  **w/ metastases** |
| --- | --- | --- |
| **Patients (n)**  Cases  Cases with available metastatic tissue | 26  0 | 26  24 |
| **Gender (n (%))**  Male  Female | 10 (38.5%)  16 (61.5%) | 12 (46.2%)  14 (53.8%) |
| **Age (years)**  Min-Max (Average) | 45-76 (62.1) | 38-76 (61.2) |
| **Smoking status (n (%))**  Current Smoker  Previous Smoker  Unknown | 18 (69.2%)  8 (30.8%)  0 (0%) | 16 (61.5%)  8 (30.8%)  2 (7.7%) |
| **TNM Classification (n (%))**  TX  T1  T2  T3  T4  NX  N0  N1  N2  N3  M0  M1 | 0 (0%)  9 (34.7%)  16 (61.5%)  0 (0%)  1 (3.8%)  0 (0%)  21 (80.8%)  5 (19.2)  0 (0%)  0 (0%)  26 (100%)  0 (0%) | 1 (3.8%)  9 ((34.7%)  16 (61.5%)  0 (0%)  0 (0%)  2 (7.7%)  12 (46.2%)  3 (11.4%)  9 (34.7%)  0 (0%)  0 (0%)  26 (100%) |
| **Tumor content (%)**  Tumors, Min-Max (Average)  Metastases, Min-Max (Average) | 5-60% (27.0%) | 5-80% (39.4%)  5-90% (68.7%) |
|  |  |  |

**Supplementary Table S4: DNA methylation frequencies in metastasizing and non-metastasizing primary lung tumors.**

| **ID** | **Tumors w/o metastases** | | | | |  | **Tumors w/ metastases** | | | | |  | ***P*** |
| --- | --- | --- | --- | --- | --- | --- | --- | --- | --- | --- | --- | --- | --- |
|  | **Methylation Level** | | | | |  | **Methylation Level** | | | | |  |  |
|  | **0-1%**  n (%) | **1-10%**  n (%) | **10-50%**  n (%) | **50-100%**  n (%) | ***N***  (%) |  | **0-1%**  n (%) | **1-10%**  n (%) | **10-50%**  n (%) | **50-100%**  n (%) | ***N***  (%) |  |  |
| *OTX2* | 15  (57.7) | 11  (42.3) | 0  (0) | 0  (0) | 26  (100) |  | 10  (40.0) | 15  (60.0) | 0  (0) | 0  (0) | 25  (100) |  | 0.267 |
| *HOXD3* | 0  (0) | 2  (8.0) | 19  (76.0) | 4  (16.0) | 25  (100) |  | 0  (0) | 5  (20.0) | 15  (60.0) | 5  (20.0) | 25  (100) |  | 0.796 |
| *HOXB3/HOXB4* | 9  (37.5) | 7  (29.2) | 7  (29.2) | 1  (4.1) | 24  (100) |  | 3  (12.5) | 9  (37.5) | 12  (50.0) | 0  (0) | 24  (100) |  | 0.108 |
| *HOXD10* | 4  (15.4) | 13  (50.0) | 9  (34.6) | 0  (0) | 26  (100) |  | 1  (4.0) | 14  (56.0) | 9  (36.0) | 1  (4.0) | 25  (100) |  | 0.356 |
| Chr1(q21.1).A | 0  (0) | 7  (28.0) | 15  (60.0) | 3  (12.0) | 25  (100) |  | 0  (0) | 3  (13.0) | 17  (74.0) | 3  (13.0) | 23  (100) |  | 0.378 |
| *HIST1H3G/HIST1H2BI* | 11  (42.3) | 13  (50.0) | 2  (7.7) | 0  (0) | 26  (100) |  | 8  (30.8) | 14  (53.8) | 4  (15.4) | 0  (0) | 26  (100) |  | 0.354 |
| *GHSR* | 2  (8.7) | 2  (8.7) | 15  (65.2) | 4  (17.4) | 23  (100) |  | 0  (0) | 4  (16.0) | 14  (56.0) | 7  (28.0) | 25  (100) |  | 0.412 |
| *SIM1* | 3  (12.5) | 10  (41.7) | 11  (45.8) | 0  (0) | 24  (100) |  | 1  (4.0) | 12  (48.0) | 10  (40.0) | 2  (8.0) | 25  (100) |  | 0.491 |
| *OSR1* | 1  (4.2) | 6  (25.0) | 14  (58.3) | 3  (12.5) | 24  (100) |  | 0  (0) | 6  (25.0) | 16  (66.7) | 2  (8.3) | 24  (100) |  | 0.915 |
| Chr6(p22.1) | 10  (40.0) | 2  (8.0) | 13  (52.0) | 0  (0) | 25  (100) |  | 6  (25.0) | 11  (45.8) | 3  (12.5) | 4  (16.7) | 24  (100) |  | 0.887 |
| *HOXA3* | 0  (0) | 1  (4.0) | 21  (84.0) | 3  (12.0) | 25  (100) |  | 0  (0) | 2  (8.0) | 19  (76.0) | 4  (16.0) | 25  (100) |  | >0.999 |
| *LOC648987* | 18  (85.7) | 2  (9.5) | 1  (4.8) | 0  (0) | 21  (100) |  | 16  (66.7) | 5  (20.8) | 3  (12.5) | 0  (0) | 24  (100) |  | 0.163 |
| *HIST1H2AJ/HIST1H2BM* | 20  (80.0) | 5  (20.0) | 0  (0) | 0  (0) | 25  (100) |  | 20  (76.9) | 6  (23.1) | 0  (0) | 0  (0) | 26  (100) |  | >0.999 |
| *HOXA5* | 0  (0) | 7  (26.9) | 18  (69.2) | 1  (3.9) | 26  (100) |  | 0  (0) | 3  (11.6) | 18  (69.2) | 5  (19.2) | 26  (100) |  | 0.071 |
| *HIST1H3E* | 0  (0) | 0  (0) | 4  (16.0) | 21  (84.0) | 25  (100) |  | 0  (0) | 0  (0) | 1  (4.0) | 24  (96.0) | 25  (100) |  | 0.349 |

## MS-HRM Assays

### The technical specifications for the 18 MS-HRM assays, including genomic location **(**UCSC Genome Browser on Human Dec. 2013 (GRCh38/hg38) Assembly), primer sequences and assay-specific PCR cycling and HRM protocols are shown below. For each assay, 180 bp of regional genomic sequence (top strand) and corresponding bisulfite-modified sequence (bottom strand) is shown. The locations of the primers are indicated in red. Normalized melting profiles for the DNA methylation standards are shown for each assay in technical duplicates. The DNA methylation standards were generated as a serial dilution of fully methylated DNA into an unmethylated background. The 100% methylated standard is indicated in red, 50% methylated standard in light blue, 10% methylated standard in green, 1% methylated standard in dark blue and the 0% methylated standard in orange.

### Assay 1: *OTX2*

PCR cycling and HRM protocol for the *OTX2* MS-HRM assay; 1 cycle of 95ºC for 10 minutes. 1 cycle of 95ºC for 15 seconds, 40 cycles of 1 minute at 61ºC. 95°C for 1 minute, 55ºC for 1 minute and a melting phase from 55°C to 95°C with a temperature increase of 0.1°C/sec and 50 fluorescence acquisition points per °C. 95ºC for 1 minute. Amplicon length = 92 bp.

Genomic Location Hg38: Chr14: 56809871-56809962

GTAATAACGATCGTTGCAAAAAGAAAAATGTGATCTAGAGATGAGAGCGGTAGTGGGAGA

|||||||++||++|||:|||||||||||||||||:||||||||||||++|||||||||||

GTAATAACGATCGTTGTAAAAAGAAAAATGTGATTTAGAGATGAGAGCGGTAGTGGGAGA

GAGGCAGAGAGCGTGCTCCTGGGGGTCGTCGCTTCTGCAAAACGTCGTCGAAACGCTGCG

||||:||||||++||:|::|||||||++|++:||:||:||||++|++|++|||++:||++

GAGGTAGAGAGCGTGTTTTTGGGGGTCGTCGTTTTTGTAAAACGTCGTCGAAACGTTGCG

AATGTAATCTGGGGTGTTTTGGAAGGTTTTGTTTGTGGTTTTGTTTTTATGTCAACGCCG

||||||||:|||||||||||||||||||||||||||||||||||||||||||:||++:++

AATGTAATTTGGGGTGTTTTGGAAGGTTTTGTTTGTGGTTTTGTTTTTATGTTAACGTCG

MS-HRM Primers:

### *OTX2* F: 5’ – GAG CGG TAG TGG GAG AGA GG– 3’

*OTX2* R: 5’ – CAC CCC AAA TTA CAT TCG CAA C– 3’

### *
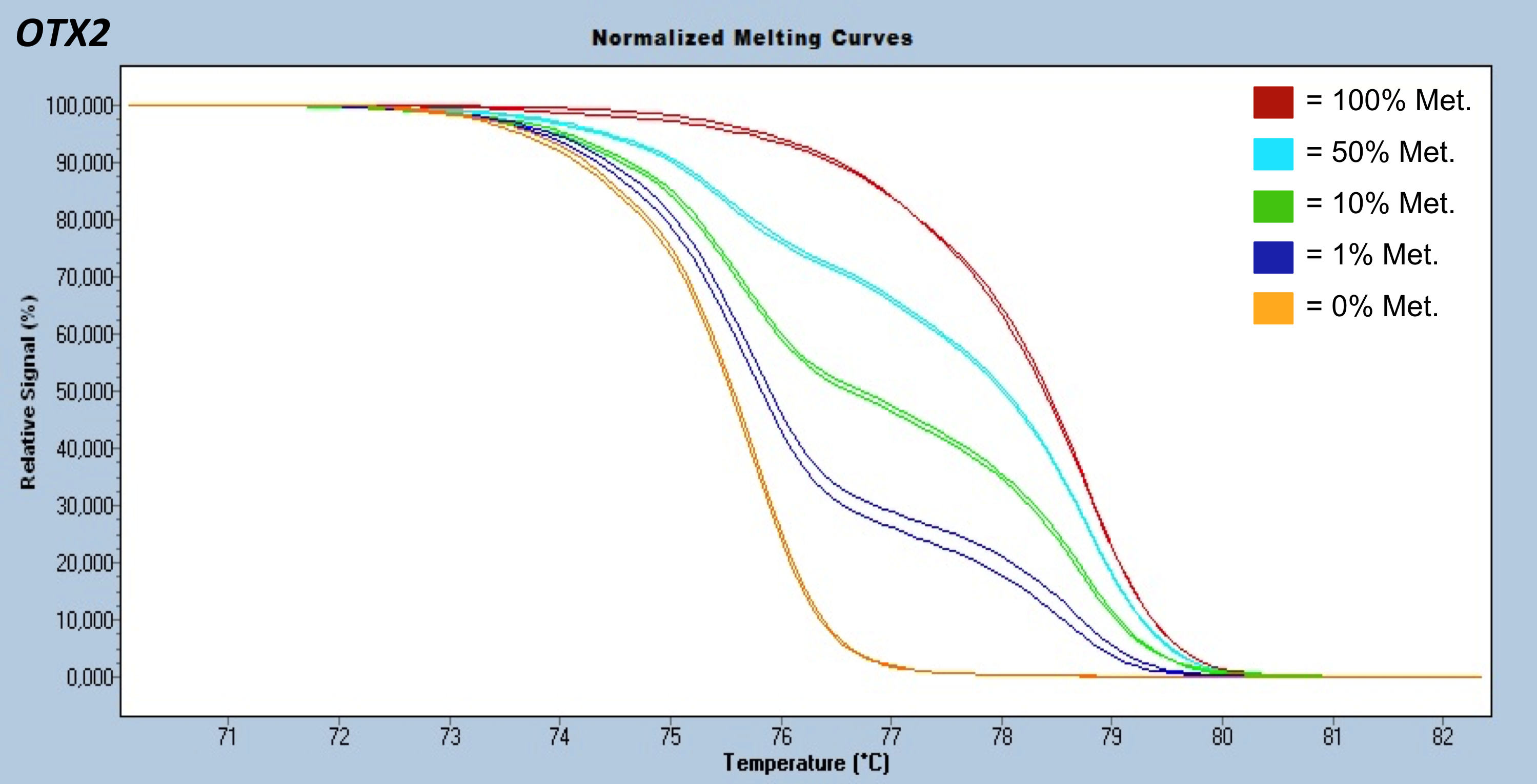
*

### Assay 2: *HOXD3*

PCR cycling and HRM protocol for the *HOXD3* MS-HRM assay; 1 cycle of 95ºC for 10 minutes. 1 cycle of 95ºC for 15 seconds, 40 cycles of 1 minute at 62ºC. 95°C for 1 minute, 55ºC for 1 minute and a melting phase from 55°C to 95°C with a temperature increase of 0.1°C/sec and 50 fluorescence acquisition points per °C. 95ºC for 1 minute. Amplicon length = 72 bp.

Genomic Location Hg38: Chr2: 176162676-176162747

ACCCGGGGCCGCCACCCCCTTATGCCTCTAGCTAGATTTTTCTTTTTCCAGCCGCGGAGG

|::++|||:++::|:::::|||||::|:|||:|||||||||:|||||::||:++++||||

ATTCGGGGTCGTTATTTTTTTATGTTTTTAGTTAGATTTTTTTTTTTTTAGTCGCGGAGG

AACAGGGTAAGTTTGCGCCTGGGGGTTCCGGGGTGCGCGGTGCGCTTTGAGCTCTTGGCG

||:||||||||||||++::||||||||:++|||||++++|||++:||||||:|:||||++

AATAGGGTAAGTTTGCGTTTGGGGGTTTCGGGGTGCGCGGTGCGTTTTGAGTTTTTGGCG

TAAGAGGCTTGGGAAGAAGAAAGGAAAGAGGACCCCAAGTTAACCAAAGTTGGACCACCA

|||||||:||||||||||||||||||||||||::::|||||||::|||||||||::|::|

TAAGAGGTTTGGGAAGAAGAAAGGAAAGAGGATTTTAAGTTAATTAAAGTTGGATTATTA

MS-HRM Primers:

### *HOXD3* F: 5’ – CGG AGG AAT AGG GTA AGT TTG– 3’

*HOXD3* R: 5’ – CTC TTA CGC CAA AAA CTC AAA AC– 3’

### *
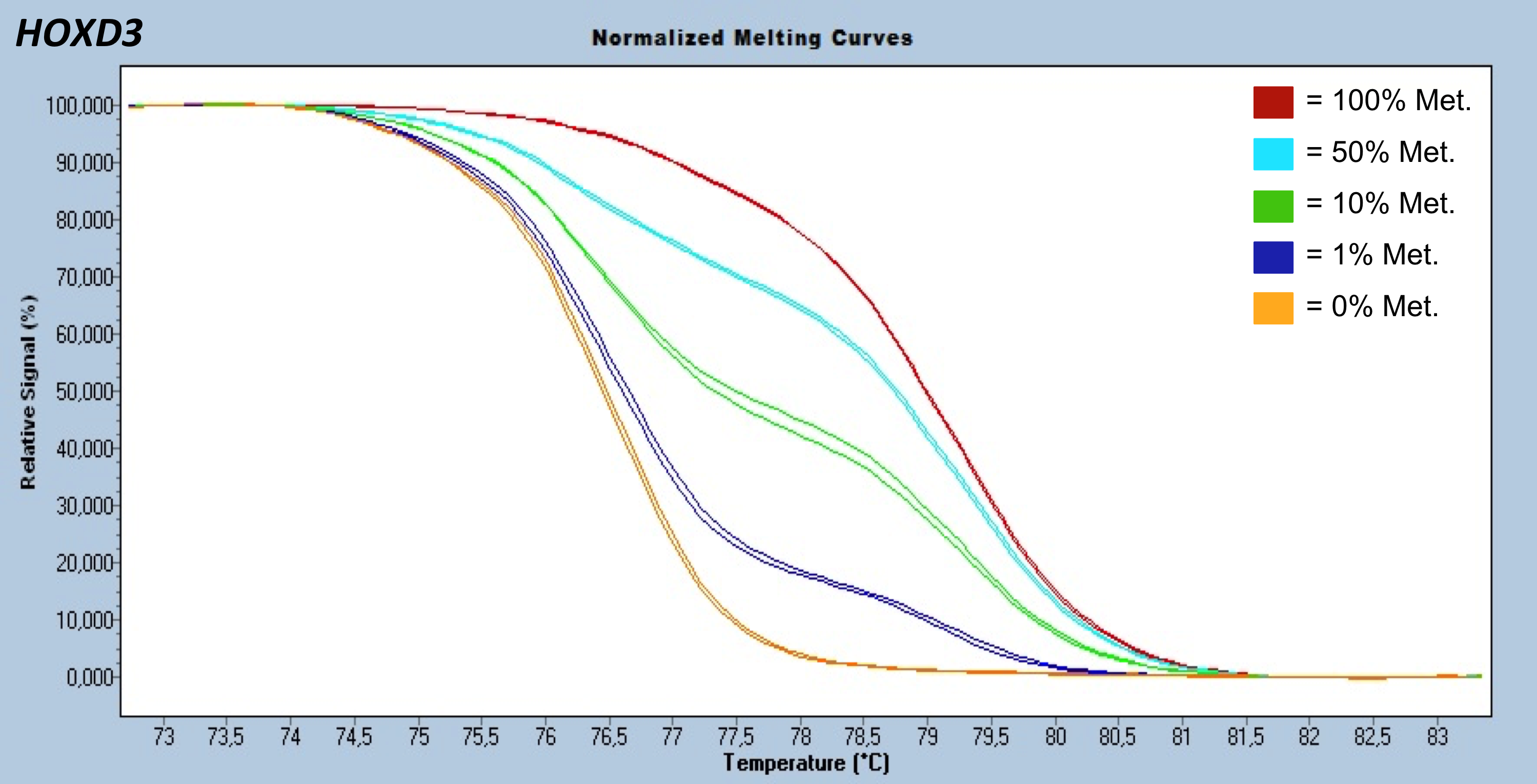
*

### Assay 3: *HOXB3/HOXB4*

PCR cycling and HRM protocol for the *HOXB3/HOXB4* MS-HRM assay; 1 cycle of 95ºC for 10 minutes. 1 cycle of 95ºC for 15 seconds, 40 cycles of 1 minute at 54ºC. 95°C for 1 minute, 55ºC for 1 minute and a melting phase from 55°C to 95°C with a temperature increase of 0.1°C/sec and 50 fluorescence acquisition points per °C. 95ºC for 1 minute. Amplicon length = 113 bp.

Genomic Location Hg38: Chr17: 48577876-48577988

GGGCCCTCCTCCCGGAGCCCGGCCAGCGCTGCGAGGCGGTCAGCAGCAGCCCCCCGCCGC

|||:::|::|::++|||::++|::||++:||++|||++||:||:||:||:::::++:++:

GGGTTTTTTTTTCGGAGTTCGGTTAGCGTTGCGAGGCGGTTAGTAGTAGTTTTTCGTCGT

CTCCCTGCGCCCAGAACCCCCTGCACCCCAGCCCGTCCCACTCCGCGTGCAAAGAGCCCG

:|:::||++:::||||:::::||:|::::||::++|:::|:|:++++||:||||||::++

TTTTTTGCGTTTAGAATTTTTTGTATTTTAGTTCGTTTTATTTCGCGTGTAAAGAGTTCG

TCGTCTACCCCTGGATGCGCAAAGTTCACGTGAGCACGGGTGAGTGCGTGGGCACCCCTT

|++|:||::::||||||++:||||||:|++||||:|++||||||||++||||:|::::||

TCGTTTATTTTTGGATGCGTAAAGTTTACGTGAGTACGGGTGAGTGCGTGGGTATTTTTT

MS-HRM Primers:

### *HOXB3/HOXB4* F: 5’ – AGG CGG TTA GTA GTA GTT T – 3’

*HOXB3/HOXB4* R: 5’ – AAC TTT ACG CAT CCA AAA ATA AAC – 3’

### *
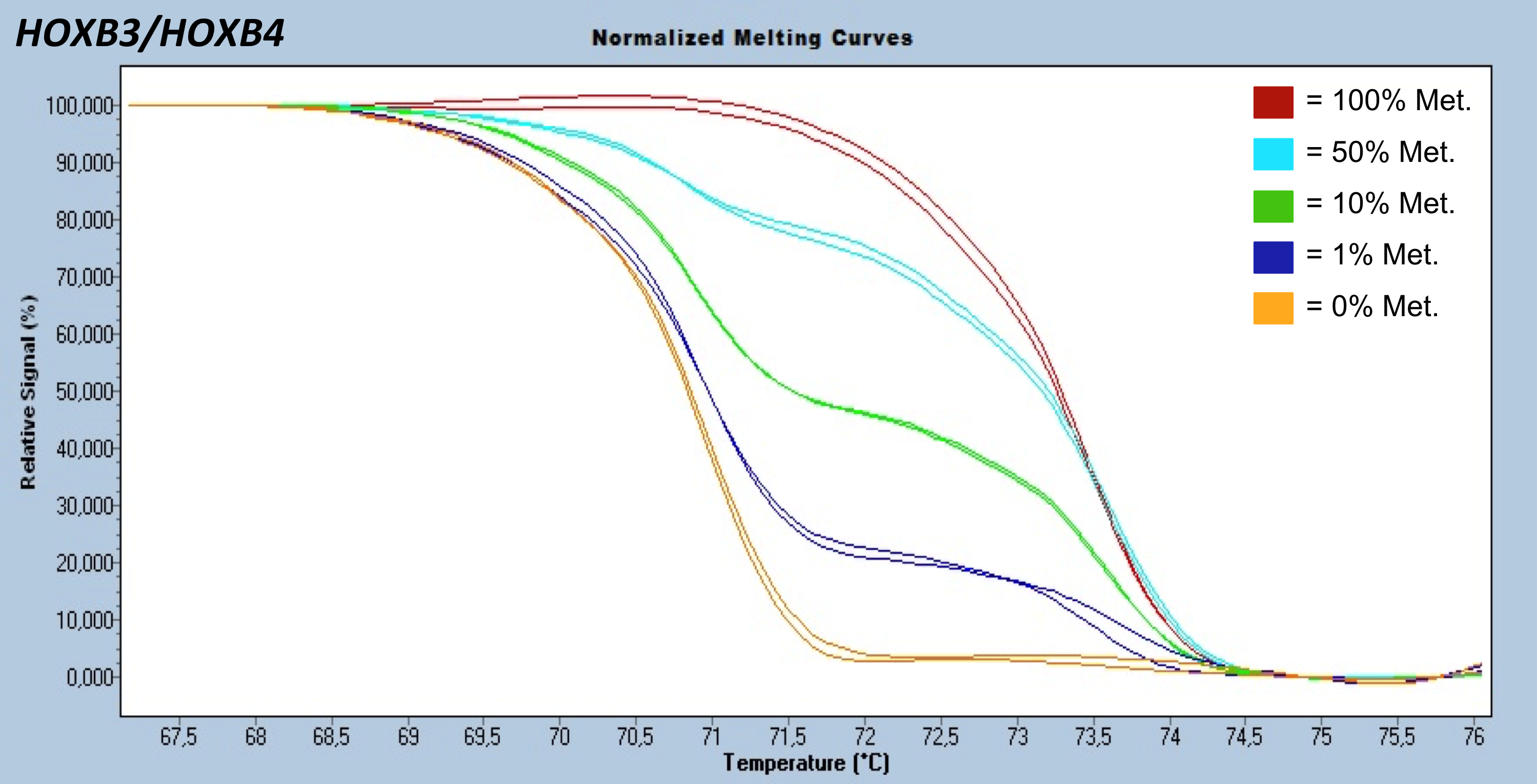
*

### Assay 4: *HOXD10*

PCR cycling and HRM protocol for the *HOXD10* MS-HRM assay; 1 cycle of 95ºC for 10 minutes.

1 cycle of 95ºC for 15 seconds, 40 cycles of 1 minute at 54ºC. 95°C for 1 minute, 55ºC for 1 minute and a melting phase from 55°C to 95°C with a temperature increase of 0.1°C/sec and 50 fluorescence acquisition points per °C. 95ºC for 1 minute. Amplicon length = 78 bp.

Genomic Location Hg38: Chr2: 176117574-176117651

CTGCCTGGCTGAGGTCTCCGTGTCCAGTCCCGAAGTGCAGGAGAAGGAAAGCAAAGGTCG :||::|||:||||||:|:++|||::|||::++|||||:|||||||||||||:||||||++

TTGTTTGGTTGAGGTTTTCGTGTTTAGTTTCGAAGTGTAGGAGAAGGAAAGTAAAGGTCG

GTATGAGCAGAGTTGCCACCCCAGCGGGGCGCGCAGCCCGGGAACCCGGCAGAGAGGGAG

|||||||:|||||||::|::::||++|||++++:||::++||||::++|:||||||||||

GTATGAGTAGAGTTGTTATTTTAGCGGGGCGCGTAGTTCGGGAATTCGGTAGAGAGGGAG

TGCCGGGGTGCCCAGCGCCGAGCCGGAGCCCGACTTGGCAGGTGCTGCTCCGCCTGGTTT

||:++|||||:::||++:++||:++|||::++|:||||:|||||:||:|:++::||||||

TGTCGGGGTGTTTAGCGTCGAGTCGGAGTTCGATTTGGTAGGTGTTGTTTCGTTTGGTTT

MS-HRM Primers:

*HOXD10* F: 5’ – TAA AGG TCG GTA TGA GTA GAG TTG TT – 3’

*hoxd10* R: 5’ – ACC CCG ACA CTC CCT CTC TA– 3’

*
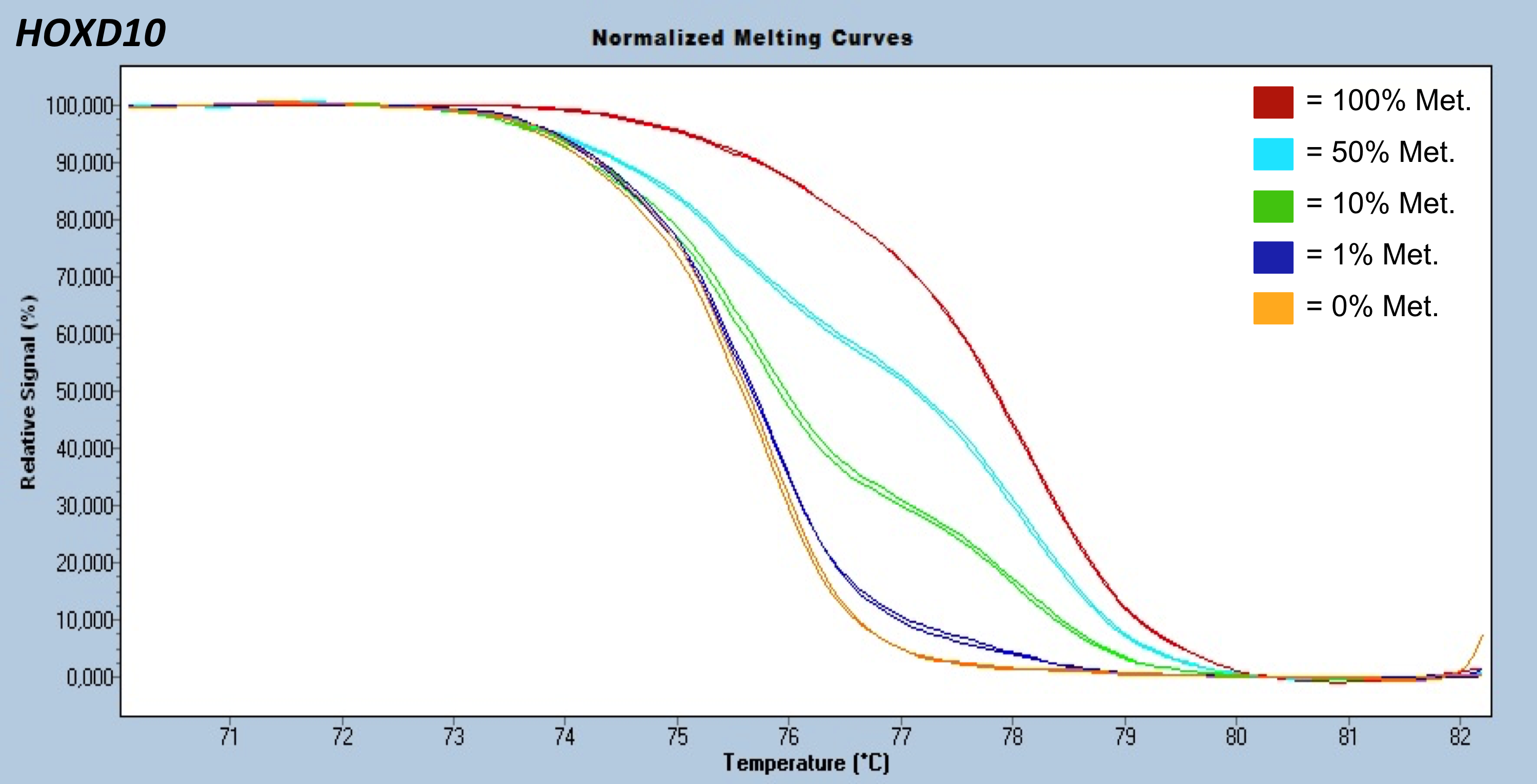
*

### Assay 5: Chr1(q21.1).A

PCR cycling and HRM protocol for the Chr1(q21.1).A MS-HRM assay; 1 cycle of 95ºC for 10 minutes. 1 cycle of 95ºC for 15 seconds, 40 cycles of 1 minute at 56ºC. 95°C for 1 minute, 55ºC for 1 minute and a melting phase from 55°C to 95°C with a temperature increase of 0.1°C/sec and 50 fluorescence acquisition points per °C. 95ºC for 1 minute. Amplicon length = 101 bp.

Genomic Location Hg38: Chr1: 147080162-147080262

CCGACTCCCGCCTCCAAGCGCGGCTCTTGGCTCGCTGGCGGGCAGCGTCCACAGAGTGTG

:++|:|::++::|::|||++++|:|:||||:|++:|||++||:||++|::|:||||||||

TCGATTTTCGTTTTTAAGCGCGGTTTTTGGTTCGTTGGCGGGTAGCGTTTATAGAGTGTG

GAACCGCCGCAGCCGCAGCTCCCGCCCGCTGGCGGGCAGACACTAGCAGGAGAAAGGACA |||:++:++:||:++:||:|::++::++:|||++||:|||:|:|||:|||||||||||:|

GAATCGTCGTAGTCGTAGTTTTCGTTCGTTGGCGGGTAGATATTAGTAGGAGAAAGGATA

CAAGGCCTGCGTGGTGGGAAAGCATGGGAGACCTCGCTTTCCCACCGGACGAGAAGGTCT

:||||::||++|||||||||||:||||||||::|++:|||:::|:++||++|||||||:|

TAAGGTTTGCGTGGTGGGAAAGTATGGGAGATTTCGTTTTTTTATCGGACGAGAAGGTTT

MS-HRM Primers:

Chr1(q21.1).A F: 5’ – GGT AGC GTT TAT AGA GTG TGG AAT– 3’

Chr1(q21.1).A R: 5’ – TTT CCC ACC ACG CAA ACC TTA TA– 3’

### *
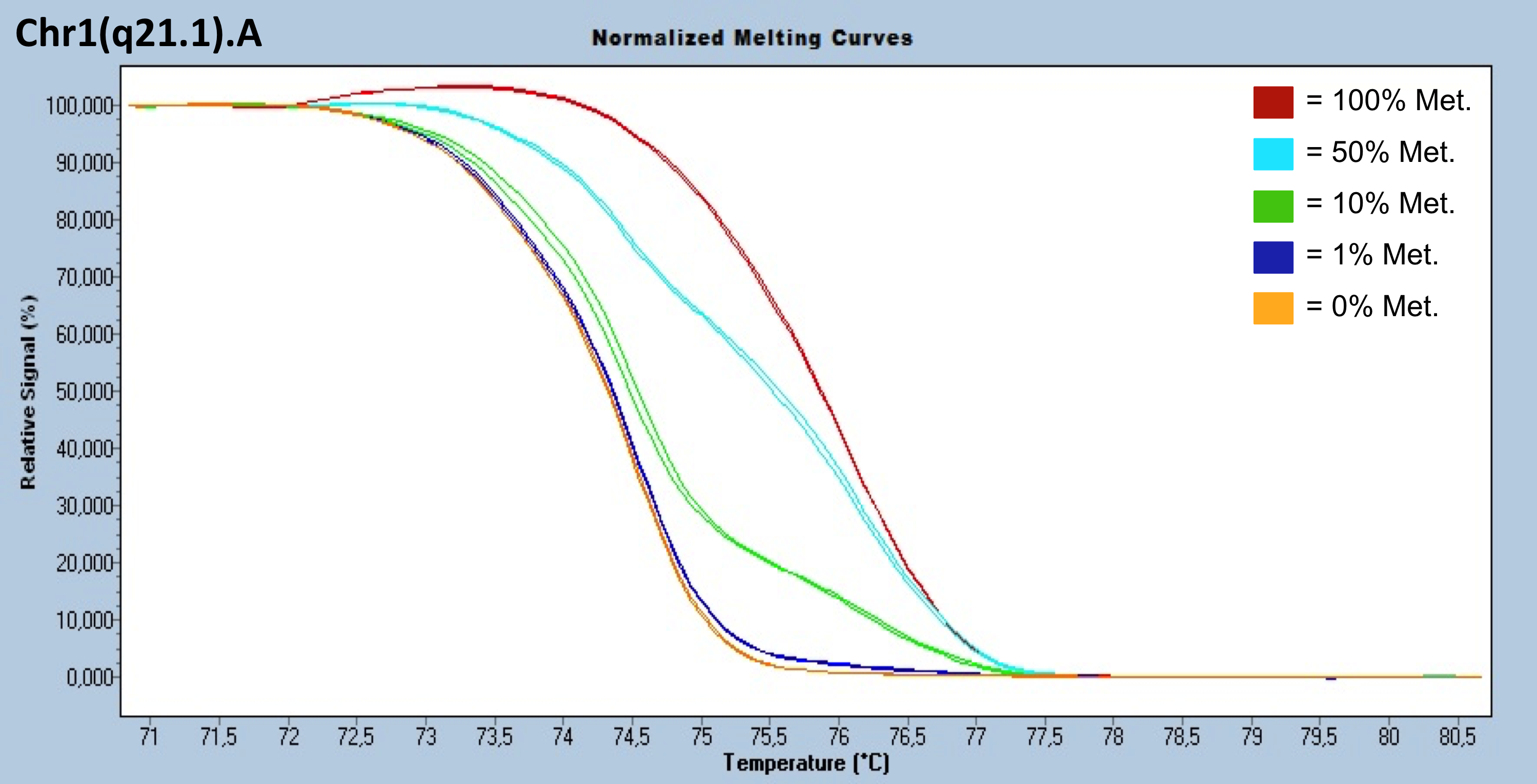
*

### Assay 6: *HIST1H3G/HIST1H2BI*

PCR cycling and HRM protocol for the *HIST1H3G/HIST1H2BI* MS-HRM assay; 1 cycle of 95ºC for 10 minutes. 1 cycle of 95ºC for 15 seconds, 40 cycles of 1 minute at 62ºC. 95°C for 1 minute, 55ºC for 1 minute and a melting phase from 55°C to 95°C with a temperature increase of 0.1°C/sec and 50 fluorescence acquisition points per °C. 95ºC for 1 minute. Amplicon length = 128 bp.

Genomic Location Hg38: Chr6: 26272252-26272379

TACCTGGAGGCGGTGCTGGAGTACCTGACCGCCAAGATCCTGGAGTTGGCGGGCAACGCG

||::||||||++|||:|||||||::|||:++::|||||::|||||||||++||:||++++

TATTTGGAGGCGGTGTTGGAGTATTTGATCGTTAAGATTTTGGAGTTGGCGGGTAACGCG

GCCCGAGACAAGAAGACCCGCGTCACCCCCCGACGCCTGCAGCTCGCCATCCACGACGAG

|::++|||:|||||||::++++|:|:::::++|++::||:||:|++::||::|++|++||

GTTCGAGATAAGAAGATTCGCGTTATTTTTCGACGTTTGTAGTTCGTTATTTACGACGAG

AAGCTCAACAAGCTGTTGGGCAAAGTTACTATCGCGCAGGGCGGTGTCCTGCCCAATATT

|||:|:||:|||:|||||||:|||||||:|||++++:||||++||||::||:::||||||

AAGTTTAATAAGTTGTTGGGTAAAGTTATTATCGCGTAGGGCGGTGTTTTGTTTAATATT

MS-HRM Primers:

### *HIST1H3G/HIST1H2BI* F: 5’ – GAT CGT TAA GAT TTT GGA GTT GG – 3’

*HIST1H3G/HIST1H2BI* R: 5’ – CGA TAA TAA CTT TAC CCA ACA ACT T – 3’

### *
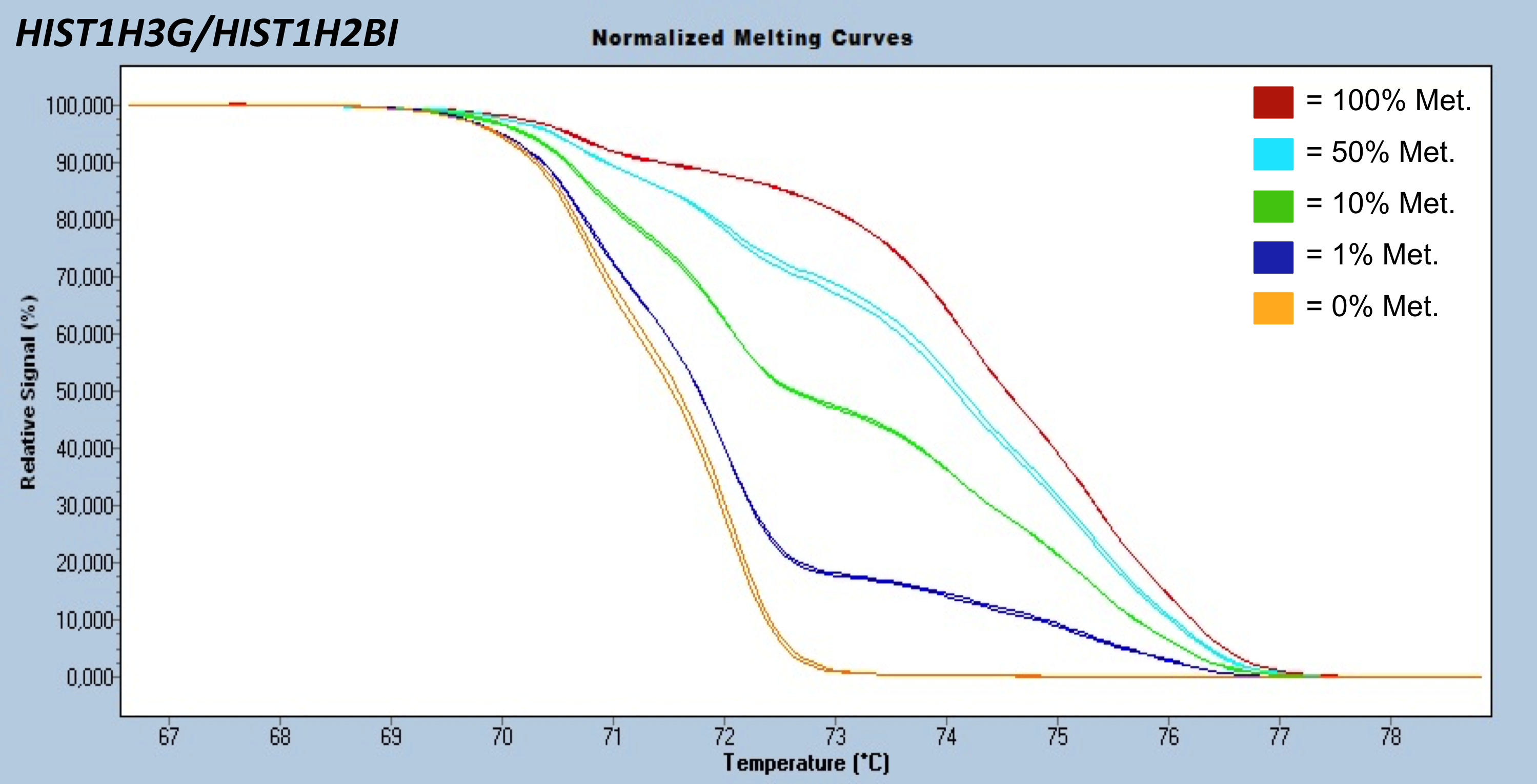
*

### Assay 7: *GHSR*

PCR cycling and HRM protocol for the *GHSR* MS-HRM assay; 1 cycle of 95ºC for 10 minutes.

1 cycle of 95ºC for 15 seconds, 40 cycles of 1 minute at 62ºC. 95°C for 1 minute, 55ºC for 1 minute and a melting phase from 55°C to 95°C with a temperature increase of 0.1°C/sec and 50 fluorescence acquisition points per °C. 95ºC for 1 minute. Amplicon length = 88 bp.

Genomic Location Hg38: Chr3: 172448360-172448447

GCTCGTCGCCCAGCGAGTCGTTGCCGGGGGAAGCATCCCAGTCCAGGTCGGCCAGTGTGA |:|++|++:::||++|||++|||:++|||||||:||:::|||::||||++|::|||||||

GTTCGTCGTTTAGCGAGTCGTTGTCGGGGGAAGTATTTTAGTTTAGGTCGGTTAGTGTGA

GGTTGAACCCCGGCTCTTCGCTGGGCGTCGCGTTCCACATGCTGCCGGCTCAGCTGAACA |||||||:::++|:|:||++:||||++|++++||::|:|||:||:++|:|:||:||||:|

GGTTGAATTTCGGTTTTTCGTTGGGCGTCGCGTTTTATATGTTGTCGGTTTAGTTGAATA

GGCTCTGGGACGTGACTGCGCTGGGAGGCTGGACCGAGCTGGCTCCCGAGGAGGTCCGCT ||:|:|||||++|||:||++:|||||||:||||:++||:|||:|::++|||||||:++:|

GGTTTTGGGACGTGATTGCGTTGGGAGGTTGGATCGAGTTGGTTTTCGAGGAGGTTCGTT

MS-HRM Primers:

*GHSR* F: 5’ – GTC GGT TAG TGT GAG GTT GAA TT– 3’

*GHSR* R: 5’ – CAC GTC CCA AAA CCT ATT CAA CTA– 3’

*
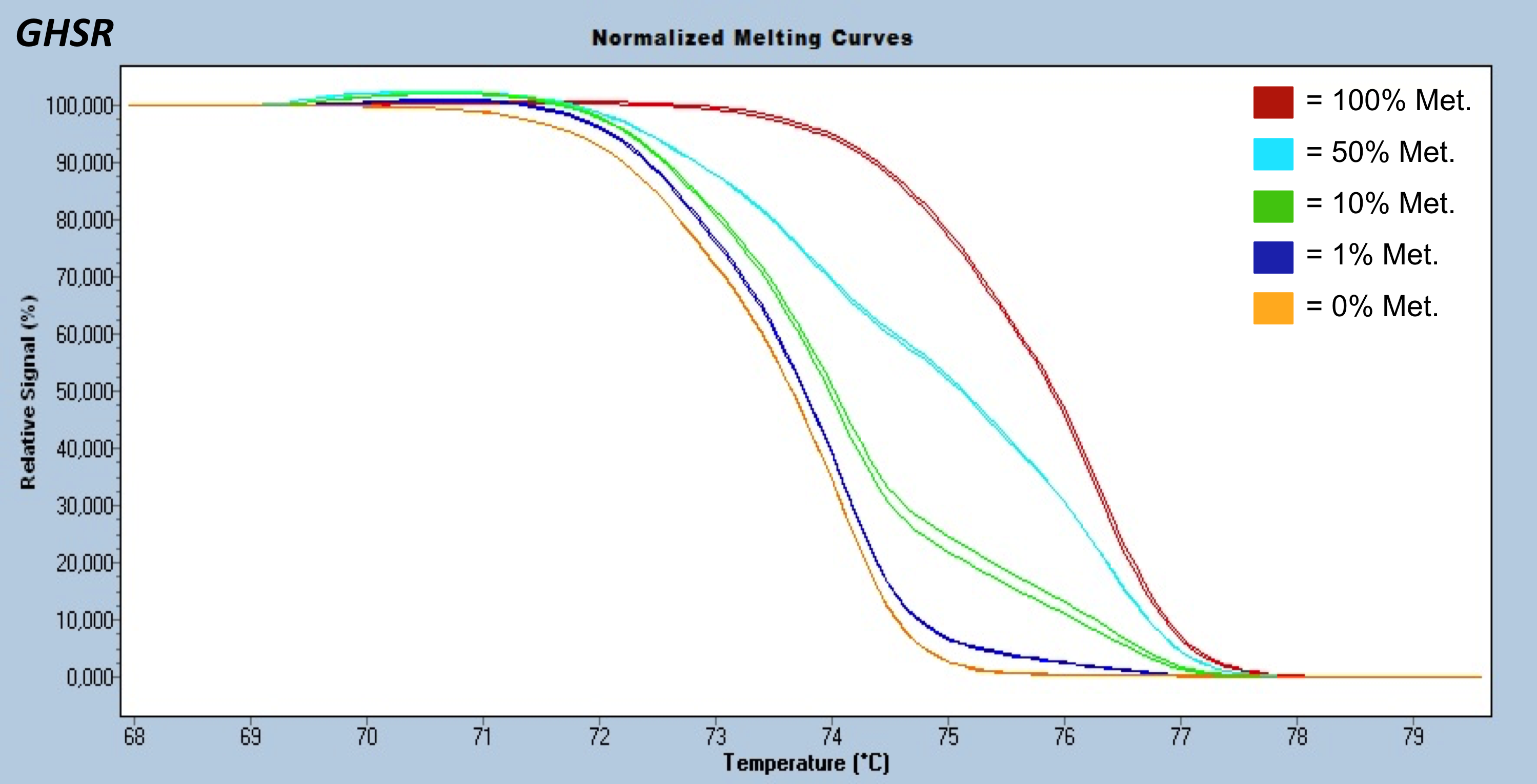
*

### Assay 8: *SIM1*

PCR cycling and HRM protocol for the *SIM1* MS-HRM assay; 1 cycle of 95ºC for 10 minutes.

1 cycle of 95ºC for 15 seconds, 40 cycles of 1 minute at 54ºC. 95°C for 1 minute, 55ºC for 1 minute and a melting phase from 55°C to 95°C with a temperature increase of 0.1°C/sec and 50 fluorescence acquisition points per °C. 95ºC for 1 minute. Amplicon length = 95 bp.

Genomic Location Hg38: Chr6: 100465031-100465125

GCCCCCTCGCCTCCCATTGGCCTGGCGCGGCCAGGGGCGAGGCGCTCATTGGCCAATAGG |:::::|++::|:::|||||::|||++++|::|||||++|||++:|:|||||::||||||

GTTTTTTCGTTTTTTATTGGTTTGGCGCGGTTAGGGGCGAGGCGTTTATTGGTTAATAGG

GCTGAGTGACACGAGTCGGCCCCGAGCCGCCCTCCGCGCGCCCAGGCTCCGGGCTCTGAA |:|||||||:|++|||++|:::++||:++:::|:++++++:::|||:|:++||:|:||||

GTTGAGTGATACGAGTCGGTTTCGAGTCGTTTTTCGCGCGTTTAGGTTTCGGGTTTTGAA

TCTTACTACCCGCGGGACCGCTGGACTCCTAATGAGCTCAGGGTGCCGGGTCGGCGCTGT

|:|||:||::++++|||:++:||||:|::|||||||:|:||||||:++|||++|++:|||

TTTTATTATTCGCGGGATCGTTGGATTTTTAATGAGTTTAGGGTGTCGGGTCGGCGTTGT

MS-HRM Primers:

*SIM1* F: 5’ – GTT TAT TGG TTA ATA GGG TTG AGT GAT – 3’

*SIM1* R: 5’ – ATC CCG CGA ATA ATA AAA TTC AAA ACC – 3’

### *
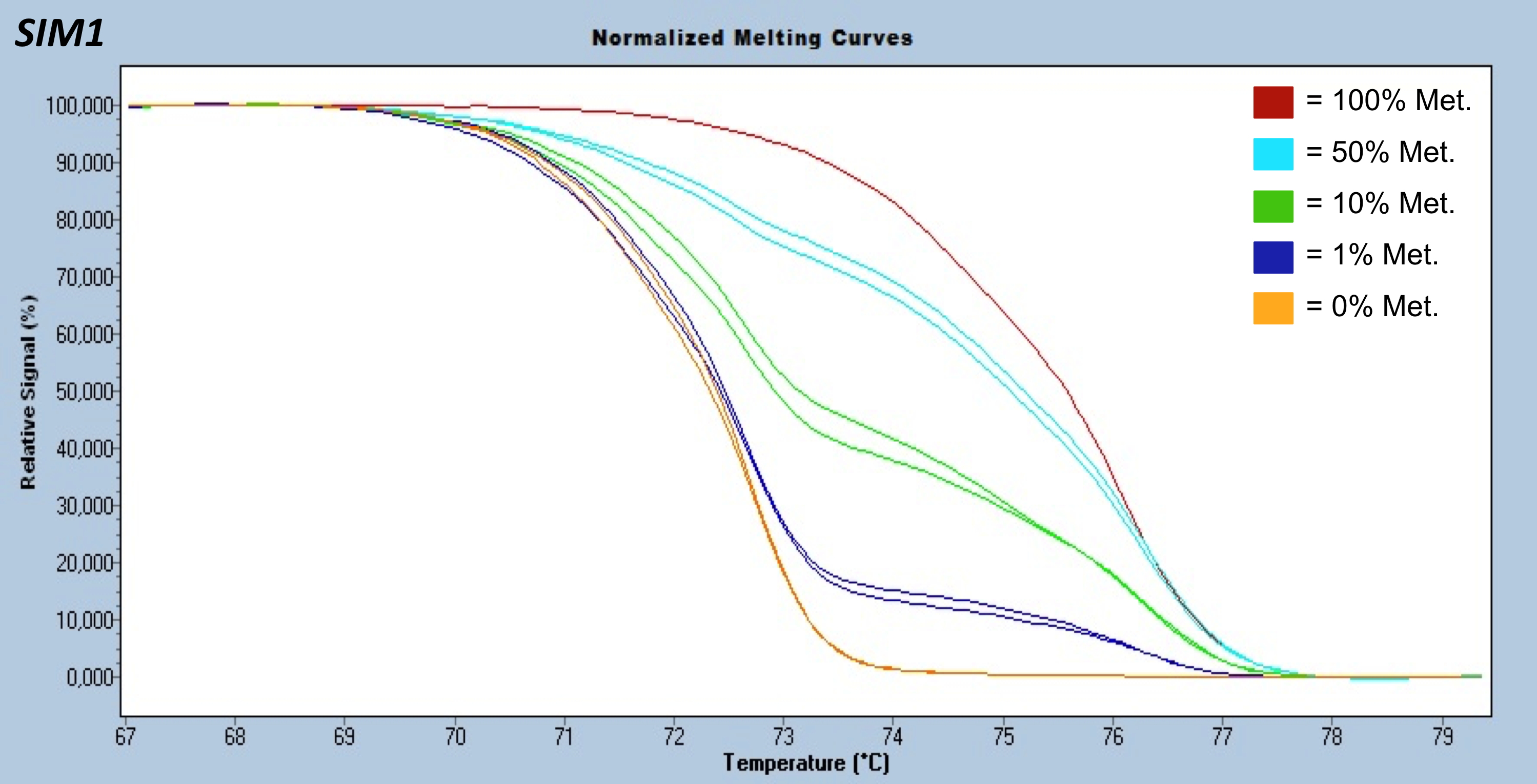
*

### Assay 9: *OSR1*

PCR cycling and HRM protocol for the *OSR1* MS-HRM assay; 1 cycle of 95ºC for 10 minutes. 1 cycle of 95ºC for 15 seconds, 40 cycles of 1 minute at 60ºC. 95°C for 1 minute, 55ºC for 1 minute and a melting phase from 55°C to 95°C with a temperature increase of 0.1°C/sec and 50 fluorescence acquisition points per °C. 95ºC for 1 minute. Amplicon length = 102 bp.

Genomic Location Hg38: Chr2: 19357150-19357251

GCTAGGTGTCCCAGGTTCACAACCCCCTAGGGAAGAGAAGCGCTGGAGGGGACTAGCAGC

|:|||||||:::|||||:|:||:::::|||||||||||||++:|||||||||:|||:||+

GTTAGGTGTTTTAGGTTTATAATTTTTTAGGGAAGAGAAGCGTTGGAGGGGATTAGTAGC

GACCGGCGGCGTGTAGATGTTCCATCCCAGGCTCCTCGAGGTAATTGCTGTTTTATTAAG

+|:++|++|++||||||||||::||:::|||:|::|++|||||||||:||||||||||||

GATCGGCGGCGTGTAGATGTTTTATTTTAGGTTTTTCGAGGTAATTGTTGTTTTATTAAG

ATTGGGGAATGGATCACCGAGGACGCAGCGGACATTTAAGTGCAGCCGGATGACCGCCTT

||||||||||||||:|:++||||++:||++||:|||||||||:||:++|||||:++::||

ATTGGGGAATGGATTATCGAGGACGTAGCGGATATTTAAGTGTAGTCGGATGATCGTTTT

MS-HRM Primers:

### *OSR1* F: 5’ – GCG TTG GAG GGG ATT AGT AG– 3’

*OSR1* R: 5’ – TCA TCC GAC TAC ACT TAA ATA TCC– 3’

### *
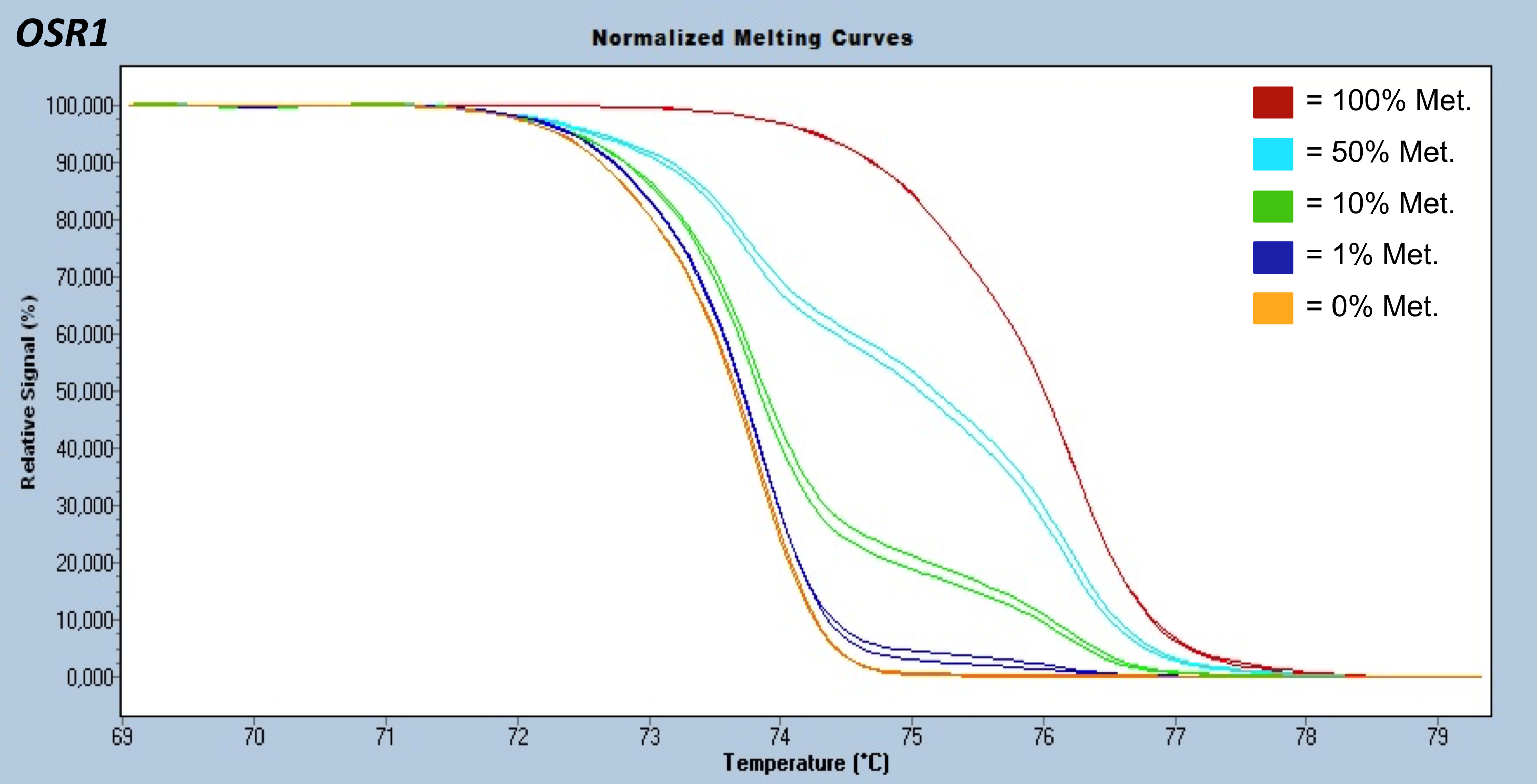
*

### Assay 10: *FRG1BP*

PCR cycling and HRM protocol for the *FRG1B* MS-HRM assay; 1 cycle of 95ºC for 10 minutes. 1 cycle of 95ºC for 15 seconds, 40 cycles of 1 minute at 61ºC. 95°C for 1 minute, 55ºC for 1 minute and a melting phase from 55°C to 95°C with a temperature increase of 0.1°C/sec and 50 fluorescence acquisition points per °C. 95ºC for 1 minute. Amplicon length = 99 bp.

Genomic Location Hg38: Chr20: 30377475-30377573

CCCCTCTCTGCACAGGCGCCAGGAACCGCGGTCCGGCCTCCGTCCAGCCCAGACAGGGTC

::::|:|:||:|:|||++::|||||:++++||:++|::|:++|::||:::|||:|||||:

TTTTTTTTTGTATAGGCGTTAGGAATCGCGGTTCGGTTTTCGTTTAGTTTAGATAGGGTT

AGAGCGAAGCCTGGGAGGCCACAAAGCCGGCTCTCTGCACCACGGCTTCCACCGGATTCG

||||++|||::|||||||::|:||||:++|:|:|:||:|::|++|:||::|:++||||++

AGAGCGAAGTTTGGGAGGTTATAAAGTCGGTTTTTTGTATTACGGTTTTTATCGGATTCG

CGGGGGTGGAGTGCATCCGAAAAGAACTGAGGAGGCTCCCACCAGAGCTGCAGGACCCAG

++|||||||||||:||:++|||||||:||||||||:|:::|::||||:||:||||:::||

CGGGGGTGGAGTGTATTCGAAAAGAATTGAGGAGGTTTTTATTAGAGTTGTAGGATTTAG

MS-HRM Primers:

### *FRG1B* F: 5’ – CGT TTA GTT TAG ATA GGG TTA G – 3’

*FRG1B* R: 5’ – CGA ATA CAC TCC ACC CCC – 3’


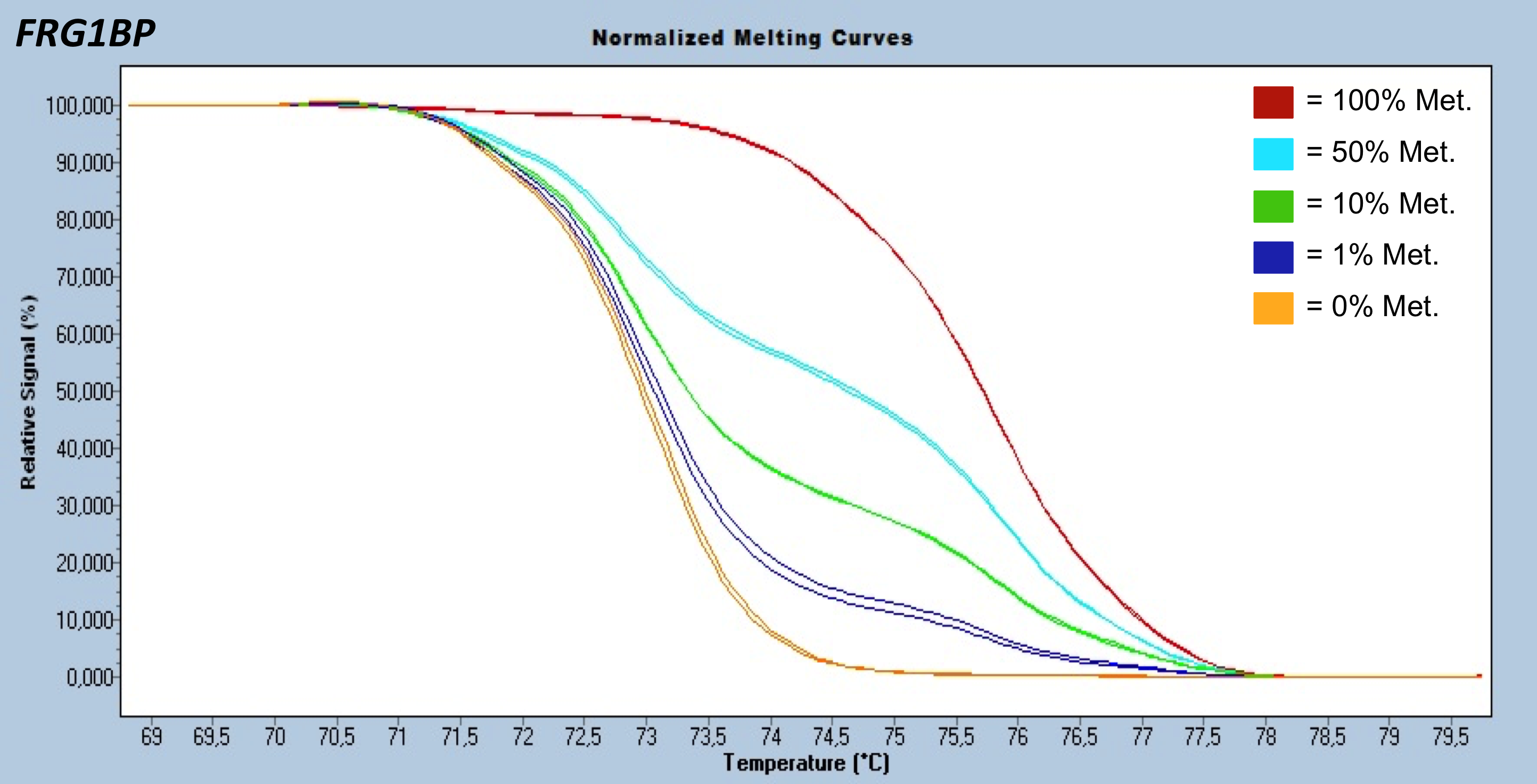


### Assay 11: Chr6(p22.1)

PCR cycling and HRM protocol for the Chr6(p22.1) MS-HRM assay; 1 cycle of 95ºC for 10 minutes. 1 cycle of 95ºC for 15 seconds, 40 cycles of 1 minute at 57ºC. 95°C for 1 minute, 55ºC for 1 minute and a melting phase from 55°C to 95°C with a temperature increase of 0.1°C/sec and 50 fluorescence acquisition points per °C. 95ºC for 1 minute. Amplicon length = 120 bp.

Genomic Location Hg38: Chr6: 28207550-28207669

CACCCAAATCAGCTGCCCCTGTTTGCAGCCACGAGGGAGTCGGGAACCAAGTCCGGACCA

:|:::||||:||:||::::||||||:||::|++|||||||++||||::||||:++||::|

TATTTAAATTAGTTGTTTTTGTTTGTAGTTACGAGGGAGTCGGGAATTAAGTTCGGATTA

CCGTCTCCTAGAGAATTACATGTCCCTGCACGCACGGAGAATAAAGCCAGGAACGATAAG

:++|:|::||||||||||:||||:::||:|++:|++||||||||||::|||||++|||||

TCGTTTTTTAGAGAATTATATGTTTTTGTACGTACGGAGAATAAAGTTAGGAACGATAAG

TGGTAAATTATAGGGAATTTGGGCGGTGGTGACATAGTTAACGCCATTCTCGCCCTGCTG

|||||||||||||||||||||||++|||||||:||||||||++::|||:|++:::||:||

TGGTAAATTATAGGGAATTTGGGCGGTGGTGATATAGTTAACGTTATTTTCGTTTTGTTG

MS-HRM Primers:

### Chr6(p22.1) F: 5’ – CGA GGG AGT CGG GAA TTA AG – 3’

Chr6(p22.1) R: 5’ – CAC CAC CGC CCA AAT TCC C – 3’

### *
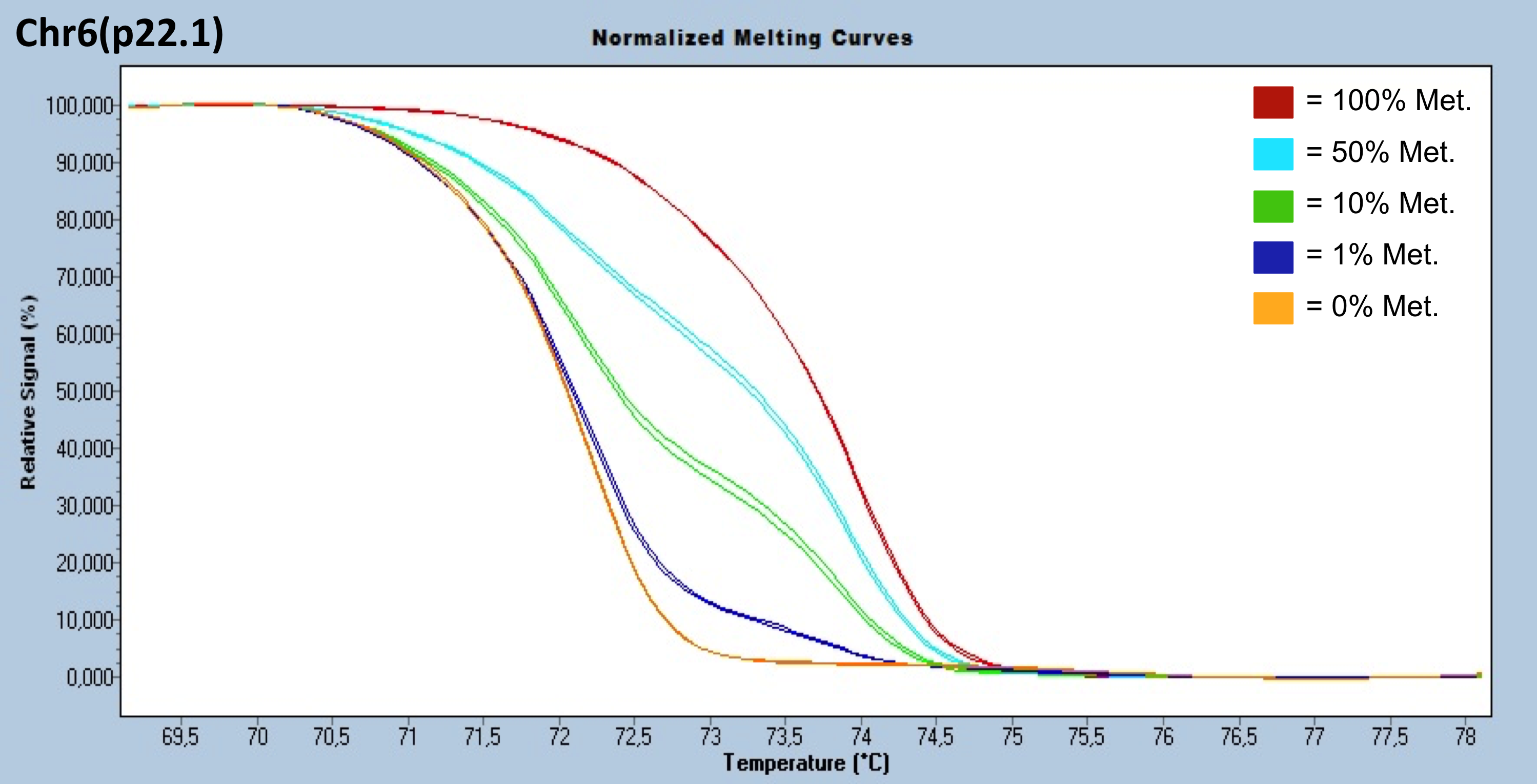
*

### Assay 12: *HOXA3*

PCR cycling and HRM protocol for the *HOXA3* MS-HRM assay; 1 cycle of 95ºC for 10 minutes. 1 cycle of 95ºC for 15 seconds, 40 cycles of 1 minute at 60ºC. 95°C for 1 minute, 55ºC for 1 minute and a melting phase from 55°C to 95°C with a temperature increase of 0.1°C/sec and 50 fluorescence acquisition points per °C. 95ºC for 1 minute. Amplicon length = 112 bp.

Genomic Location Hg38: Chr7: 27124232-27124343

ACGGATGCGCAGACAGTTGGTAATGTTCTGATTCACGCTGGGGAAGGCTGCAGAGATACC

|++||||++:|||:|||||||||||||:|||||:|++:|||||||||:||:|||||||::

ACGGATGCGTAGATAGTTGGTAATGTTTTGATTTACGTTGGGGAAGGTTGTAGAGATATT

ACAGGACGGGCGCGCGGCTTTGTTCAATTTTCCCGGCGTTCATAAATCACCCGCGCCGGG

|:||||++||++++++|:||||||:||||||::++|++||:||||||:|::++++:++||

ATAGGACGGGCGCGCGGTTTTGTTTAATTTTTTCGGCGTTTATAAATTATTCGCGTCGGG

CGAGCGAGGGAGCAAGCGAGCGCCAAAAACGCGGAGAGAGAGGCCACGGCGGCGGCGGCA

++||++||||||:|||++||++::|||||++++||||||||||::|++|++|++|++|:|

CGAGCGAGGGAGTAAGCGAGCGTTAAAAACGCGGAGAGAGAGGTTACGGCGGCGGCGGTA

MS-HRM Primers:

### *HOXA3* F: 5’ – ACG TTG GGG AAG GTT GTA GAG– 3’

*HOXA3* R: 5’ – TTA ACG CTC GCT TAC TCC CTC– 3’

### *
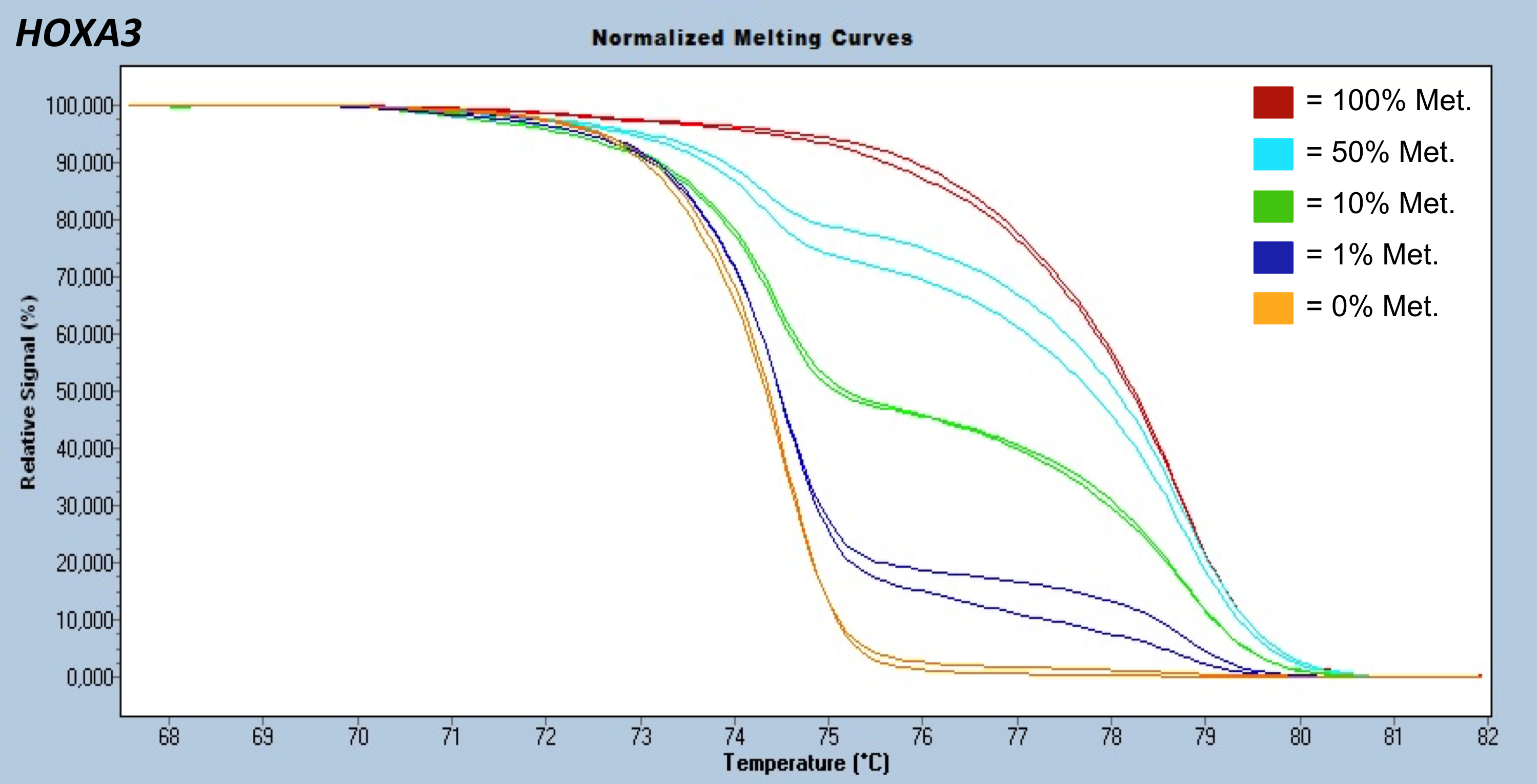
*

### Assay 13: *LY75-CD302*

PCR cycling and HRM protocol for the *LY75-CD302* MS-HRM assay; 1 cycle of 95ºC for 10 minutes. 1 cycle of 95ºC for 15 seconds, 40 cycles of 1 minute at 62ºC. 95°C for 1 minute, 55ºC for 1 minute and a melting phase from 55°C to 95°C with a temperature increase of 0.1°C/sec and 50 fluorescence acquisition points per °C. 95ºC for 1 minute. Amplicon length = 80 bp.

Genomic Location Hg38: Chr2: 159797756-159797835

TCCTCACCGCAGACCCCACTCCGCGGGGAGGGAACCCCCAAATTAGGCCAGTTGGCCGGA

|::|:|:++:|||::::|:|:++++|||||||||:::::||||||||::||||||:++||

TTTTTATCGTAGATTTTATTTCGCGGGGAGGGAATTTTTAAATTAGGTTAGTTGGTCGGA

GAACTGAGGGACTTGGAGTCGCACGACGGGCGCCGTTTCAGGGCAATTTCGGGCTGAAAT

|||:|||||||:|||||||++:|++|++||++:++|||:||||:|||||++||:||||||

GAATTGAGGGATTTGGAGTCGTACGACGGGCGTCGTTTTAGGGTAATTTCGGGTTGAAAT

GAGAAGCGGGGACGTTGGTGGCGATTTCCCCTGCTGGTGCGCGGCCGGAGTGGGGTTGCT

||||||++||||++|||||||++||||::::||:|||||++++|:++|||||||||||:|

GAGAAGCGGGGACGTTGGTGGCGATTTTTTTTGTTGGTGCGCGGTCGGAGTGGGGTTGTT

MS-HRM Primers:

### *LY75-CD302* F: 5’ – GTT GGT CGG AGA ATT GAG GGA– 3’

*LY75-CD302* R: 5’ – CCC GCT TCT CAT TTC AAC CC– 3’

### *
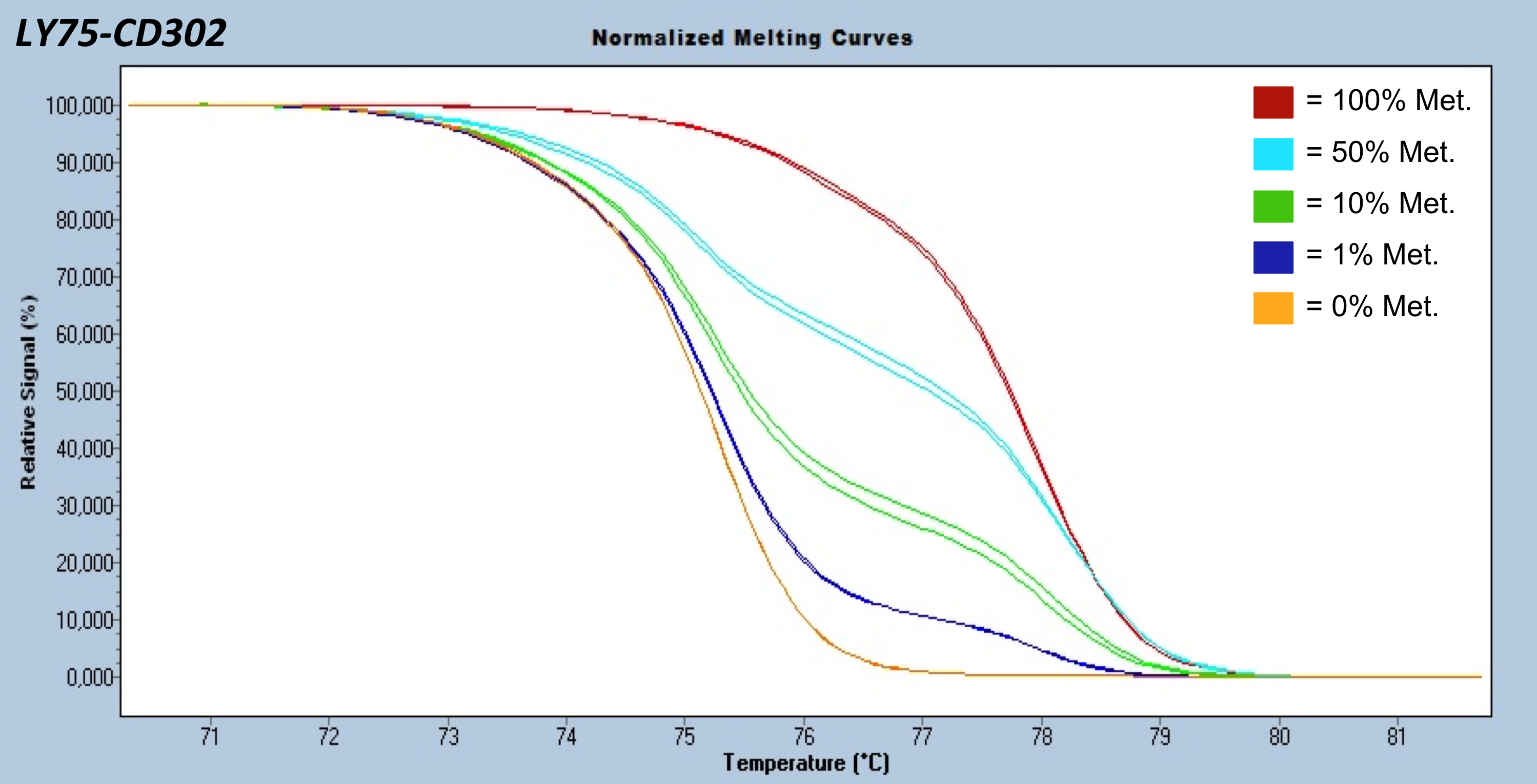
*

### Assay 14: *CTAGE15*

PCR cycling and HRM protocol for the *CTAGE15* MS-HRM assay; 1 cycle of 95ºC for 10 minutes. 1 cycle of 95ºC for 15 seconds, 40 cycles of 1 minute at 56ºC. 95°C for 1 minute, 55ºC for 1 minute and a melting phase from 55°C to 95°C with a temperature increase of 0.1°C/sec and 50 fluorescence acquisition points per °C. 95ºC for 1 minute. Amplicon length = 113 bp.

Genomic Location Hg38: Chr7: 143571685-143571797

CCGGGCTCCTCCATAGCGTCGAGGCTGCTCTGGCGGTCACCGCAGTAACACTGGCCACAA

:++||:|::|::||||++|++|||:||:|:|||++||:|:++:|||||:|:|||::|:||

TCGGGTTTTTTTATAGCGTCGAGGTTGTTTTGGCGGTTATCGTAGTAATATTGGTTATAA

CAAGCGGTGGAGAACACGCAGCCTTGGGTCTGGAACCCGAATGCGCACGTGACAACCAAC

:|||++||||||||:|++:||::||||||:|||||::++||||++:|++|||:||::||:

TAAGCGGTGGAGAATACGTAGTTTTGGGTTTGGAATTCGAATGCGTACGTGATAATTAAT

CGGAGCGGACCACTGTGGAGCGGGCTGCGGGGGGAGCTGGGGAACGCGGGCACCCACAGG

++|||++||::|:|||||||++||:||++|||||||:|||||||++++||:|:::|:|||

CGGAGCGGATTATTGTGGAGCGGGTTGCGGGGGGAGTTGGGGAACGCGGGTATTTATAGG

MS-HRM Primers:

### *CTAGE15* F: 5’ – GGT TAT CGT AGT AAT ATT GGT TAT A – 3’

*CTAGE15* R: 5’ – CAA CCC GCT CCA CAA TAA TTC – 3’

### *
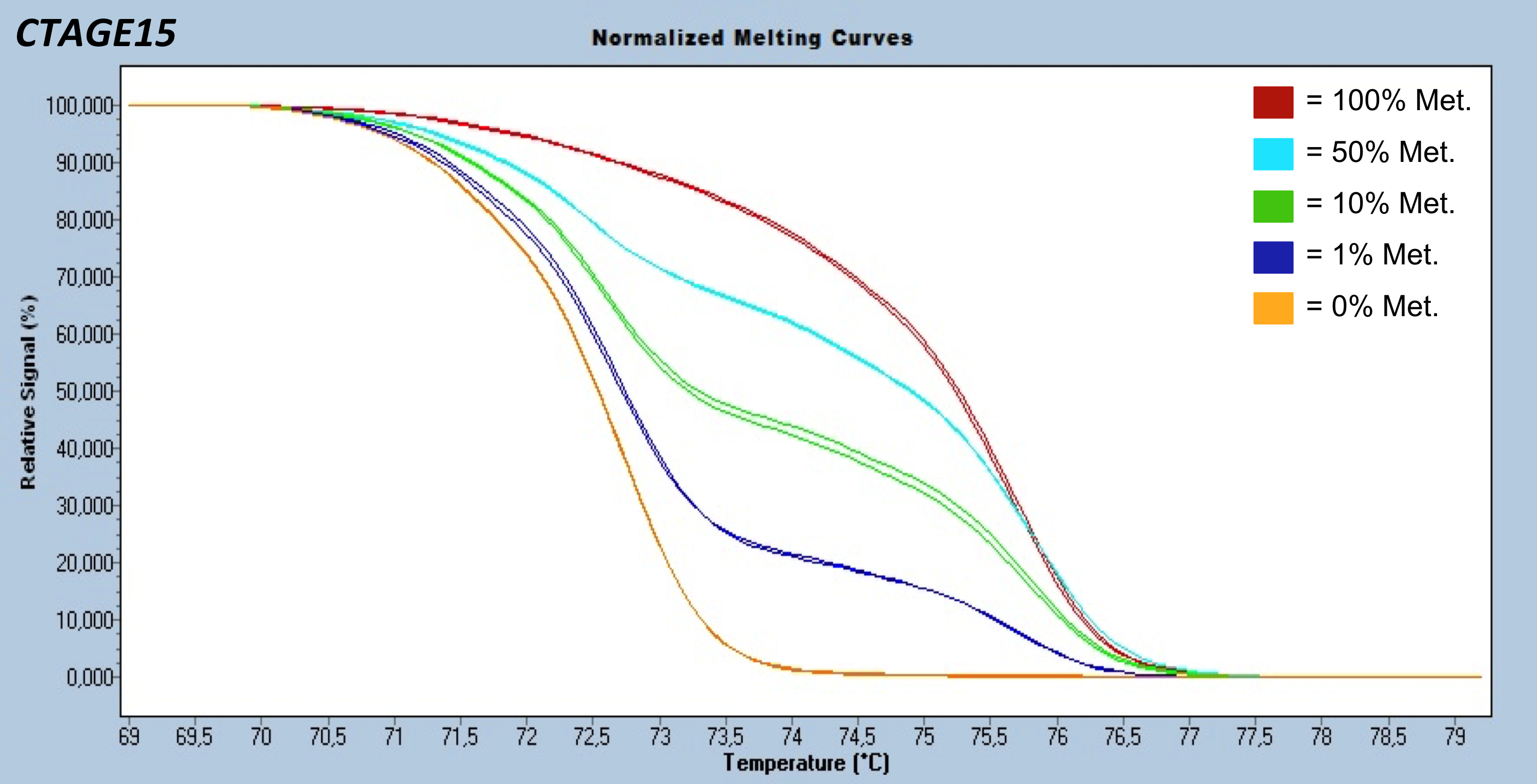
*

### Assay 15: *LOC648987*

PCR cycling and HRM protocol for the *LOC648987* MS-HRM assay; 1 cycle of 95ºC for 10 minutes. 1 cycle of 95ºC for 15 seconds, 40 cycles of 1 minute at 60ºC. 95°C for 1 minute, 55ºC for 1 minute and a melting phase from 55°C to 95°C with a temperature increase of 0.1°C/sec and 50 fluorescence acquisition points per °C. 95ºC for 1 minute. Amplicon length = 126 bp.

Genomic Location Hg38: Chr5: 43040396-43040521

TTTAGCAGCACCCTTTACGGCGCCAAAACGGATATTTGTTTGGCAATACCAGCGCTATCC

|||||:||:|:::||||++|++::||||++|||||||||||||:||||::||++:|||:+

TTTAGTAGTATTTTTTACGGCGTTAAAACGGATATTTGTTTGGTAATATTAGCGTTATTC

GCTAGGTGCCGGCGCTTGCTAAGTTCAACGCGCCAGTTTCTCGTTTGCAAGGTGGTTAGG

+:||||||:++|++:|||:||||||:||++++::|||||:|++||||:||||||||||||

GTTAGGTGTCGGCGTTTGTTAAGTTTAACGCGTTAGTTTTTCGTTTGTAAGGTGGTTAGG

GCAGAGCCCTAGCAGACAGTTTTCCGGTGGCAGCAACGCTCATTTCCCGGAAACGGGTGG

|:||||:::|||:|||:||||||:++||||:||:||++:|:||||::++||||++|||||

GTAGAGTTTTAGTAGATAGTTTTTCGGTGGTAGTAACGTTTATTTTTCGGAAACGGGTGG

MS-HRM Primers:

### *LOC648987* F: 5’ – ACG GAT ATT TGT TTG GTA ATA TTA G – 3’

*LOC648987* R: 5’ – CTA CCA CCG AAA AAC TAT CTA C – 3’

### *
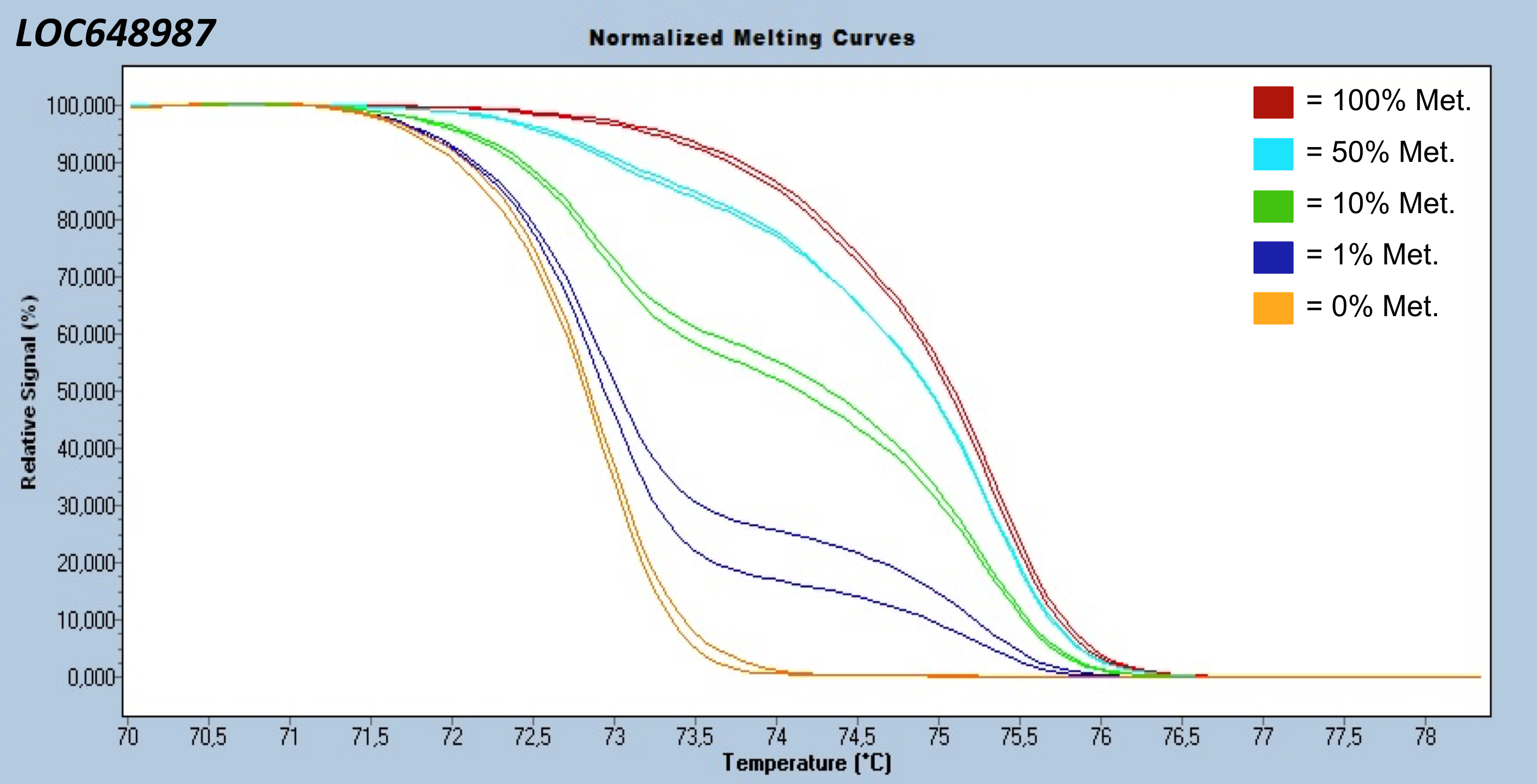
*

### Assay 16: *HIST1H2AJ/HIST1H2BM*

PCR cycling and HRM protocol for the *HIST1H2AJ/HIST1H2BM* MS-HRM assay; 1 cycle of 95ºC for 10 minutes. 1 cycle of 95ºC for 15 seconds, 40 cycles of 1 minute at 58ºC. 95°C for 1 minute, 55ºC for 1 minute and a melting phase from 55°C to 95°C with a temperature increase of 0.1°C/sec and 50 fluorescence acquisition points per °C. 95ºC for 1 minute. Amplicon length = 156 bp.

Genomic Location Hg38: Chr6: 27814577-27814732

TGCCATGTCTGGGCGTGGTAAGCAGGGAGGCAAAGCTCGCGCCAAGGCCAAGACCCGCTC

||::||||:||||++|||||||:|||||||:||||:|++++::||||::||||::++:|:

TGTTATGTTTGGGCGTGGTAAGTAGGGAGGTAAAGTTCGCGTTAAGGTTAAGATTCGTTT

TTCTCGGGCCGGGCTTCAGTTTCCCGTAGGCCGAGTGCATCGCCTGCTCCGCAAAGGCAA

||:|++||:++||:||:|||||::++||||:++||||:||++::||:|:++:|||||:||

TTTTCGGGTCGGGTTTTAGTTTTTCGTAGGTCGAGTGTATCGTTTGTTTCGTAAAGGTAA

CTATGCGGAGCGGGTCGGTGCTGGAGCGCCGGTGTACCTGGCGGCGGTGCTGGAGTACCT

:||||++|||++|||++|||:|||||++:++|||||::|||++|++|||:|||||||::|

TTATGCGGAGCGGGTCGGTGTTGGAGCGTCGGTGTATTTGGCGGCGGTGTTGGAGTATTT

MS-HRM Primers:

### *HIST1H2AJ/HIST1H2BM* F: 5’ – GCG TGG TAA GTA GGG AGG TAA AG – 3’

*HIST1H2AJ/HIST1H2BM* R: 5’ – ACC GAC GCT CCA ACA CC – 3’

### *
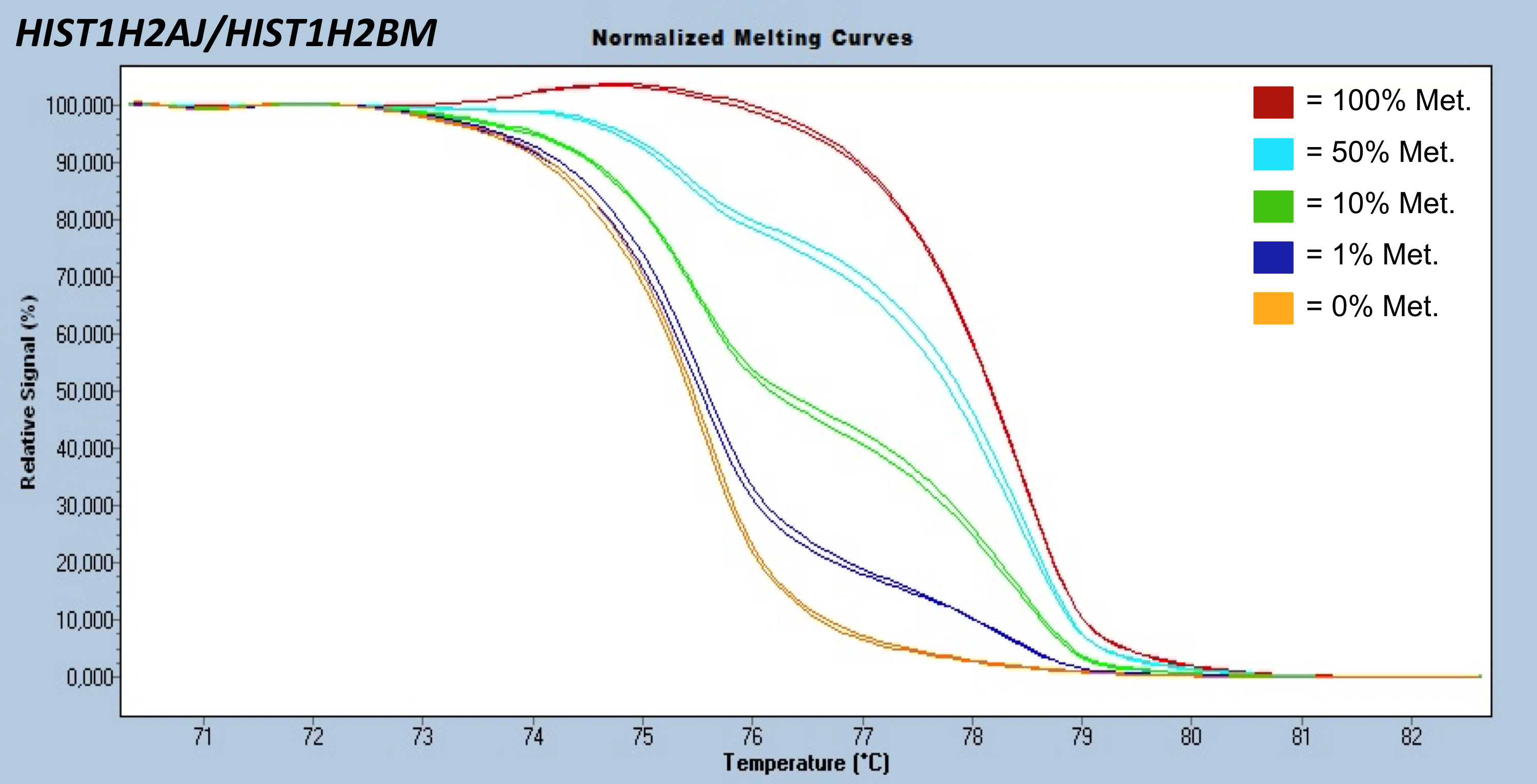
*

### Assay 17: *HOXA5*

PCR cycling and HRM protocol for the *HOXA5* MS-HRM assay; 1 cycle of 95ºC for 10 minutes. 1 cycle of 95ºC for 15 seconds, 40 cycles of 1 minute at 54ºC. 95°C for 1 minute, 55ºC for 1 minute and a melting phase from 55°C to 95°C with a temperature increase of 0.1°C/sec and 50 fluorescence acquisition points per °C. 95ºC for 1 minute. Amplicon length = 90 bp.

Genomic Location Hg38: Chr7: 27143474-27143563

CACACATATCAAAAAACAAATGAGCTCTTATTTTGTAAACTCATTTTGCGGTCGCTATCC

:|:|:||||:||||||:|||||||:|:||||||||||||:|:||||||++||++:|||::

TATATATATTAAAAAATAAATGAGTTTTTATTTTGTAAATTTATTTTGCGGTCGTTATTT

AAATGGCCCGGACTACCAGTTGCATAATTATGGAGATCATAGTTCCGTGAGCGAGCAATT

||||||::++||:||::|||||:||||||||||||||:||||||:++||||++||:||||

AAATGGTTCGGATTATTAGTTGTATAATTATGGAGATTATAGTTTCGTGAGCGAGTAATT

CAGGGACTCGGCGAGCATGCACTCCGGCAGGTACGGCTACGGCTACAATGGCATGGATCT

:|||||:|++|++||:|||:|:|:++|:|||||++|:||++|:||:|||||:||||||:|

TAGGGATTCGGCGAGTATGTATTTCGGTAGGTACGGTTACGGTTATAATGGTATGGATTT

MS-HRM Primers:

### *HOXA5* F: 5’ – TGG TTC GGA TTA TTA GTT GTA TAA T – 3’

*HOXA5* R: 5’ – TAC CTA CCG AAA TAC ATA CTC – 3’


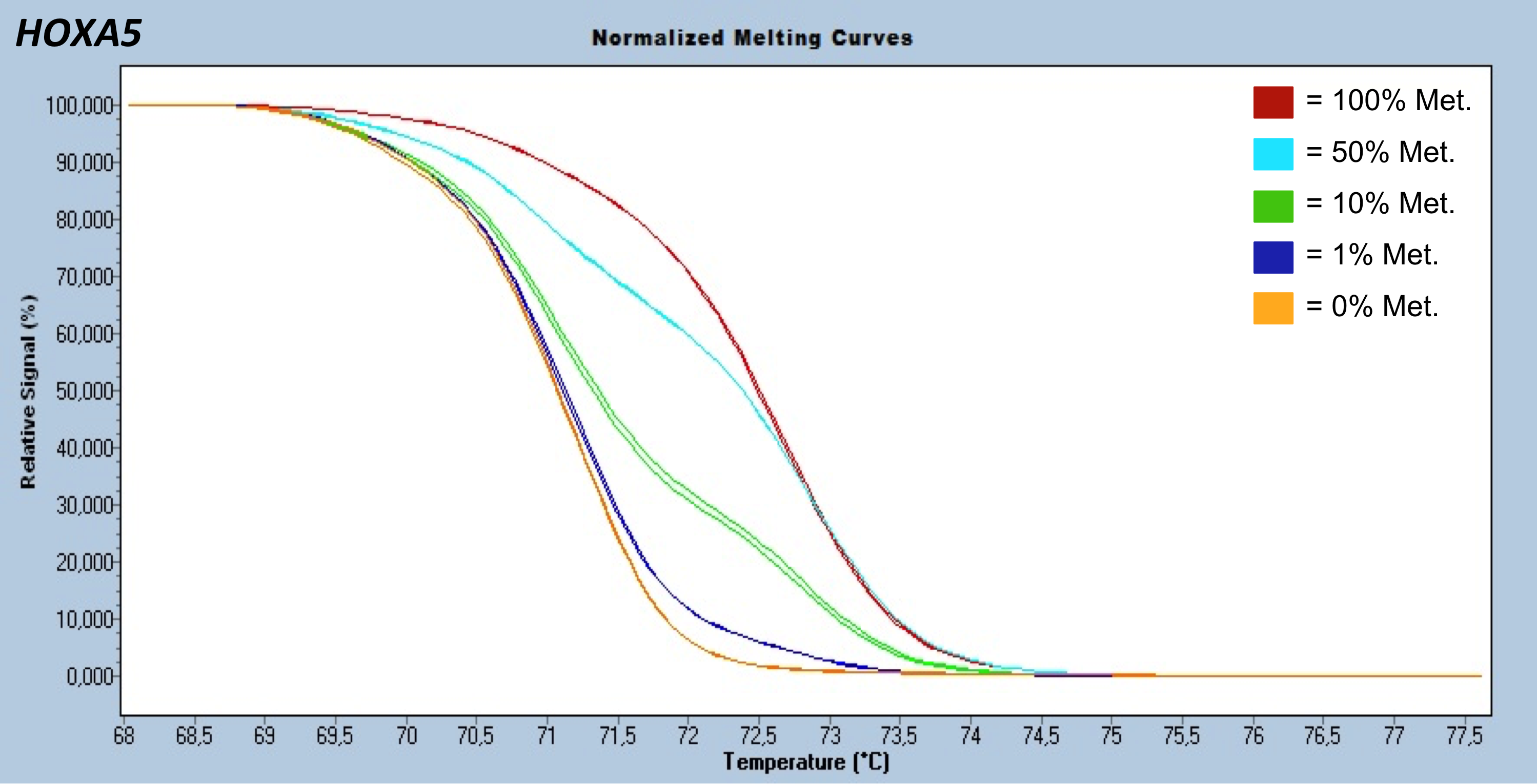


### Assay 18: *HIST1H3E*

PCR cycling and HRM protocol for the *HIST1H3E* MS-HRM assay; 1 cycle of 95ºC for 10 minutes. 1 cycle of 95ºC for 15 seconds, 40 cycles of 1 minute at 56ºC. 95°C for 1 minute, 55ºC for 1 minute and a melting phase from 55°C to 95°C with a temperature increase of 0.1°C/sec and 50 fluorescence acquisition points per °C. 95ºC for 1 minute. Amplicon length = 147 bp.

Genomic Location Hg38: Chr6: 26225275-26225421

TCCTGCAGCGCCATCACCGCGGAACTCTGGAAGCGCAGGTCGGTCTTGAAGTCCTGAGCT

|::||:||++::||:|:++++|||:|:||||||++:||||++||:|||||||::||||:|

TTTTGTAGCGTTATTATCGCGGAATTTTGGAAGCGTAGGTCGGTTTTGAAGTTTTGAGTT

ATTTCTCGCACCAGGCGCTGAAACGGCAGCTTCCGGATTAGAAGCTCGGTAGACTTCTGG

||||:|++:|::|||++:|||||++|:||:||:++|||||||||:|++|||||:||:|||

ATTTTTCGTATTAGGCGTTGAAACGGTAGTTTTCGGATTAGAAGTTCGGTAGATTTTTGG

TAGCGACGGATCTCGCGCAGAGCCACGGTGCCAGGGCGGTAGCGATGGGGCTTCTTCACG

|||++|++|||:|++++:||||::|++|||::||||++||||++||||||:||:||:|++

TAGCGACGGATTTCGCGTAGAGTTACGGTGTTAGGGCGGTAGCGATGGGGTTTTTTTACG

MS-HRM Primers:

### *HIST1H3E* F: 5’ – CGC GGA ATT TTG GAA GCG TAG G – 3’

*HIST1H3E* R: 5’ – CGC TAC CGC CCT AAC ACC – 3’


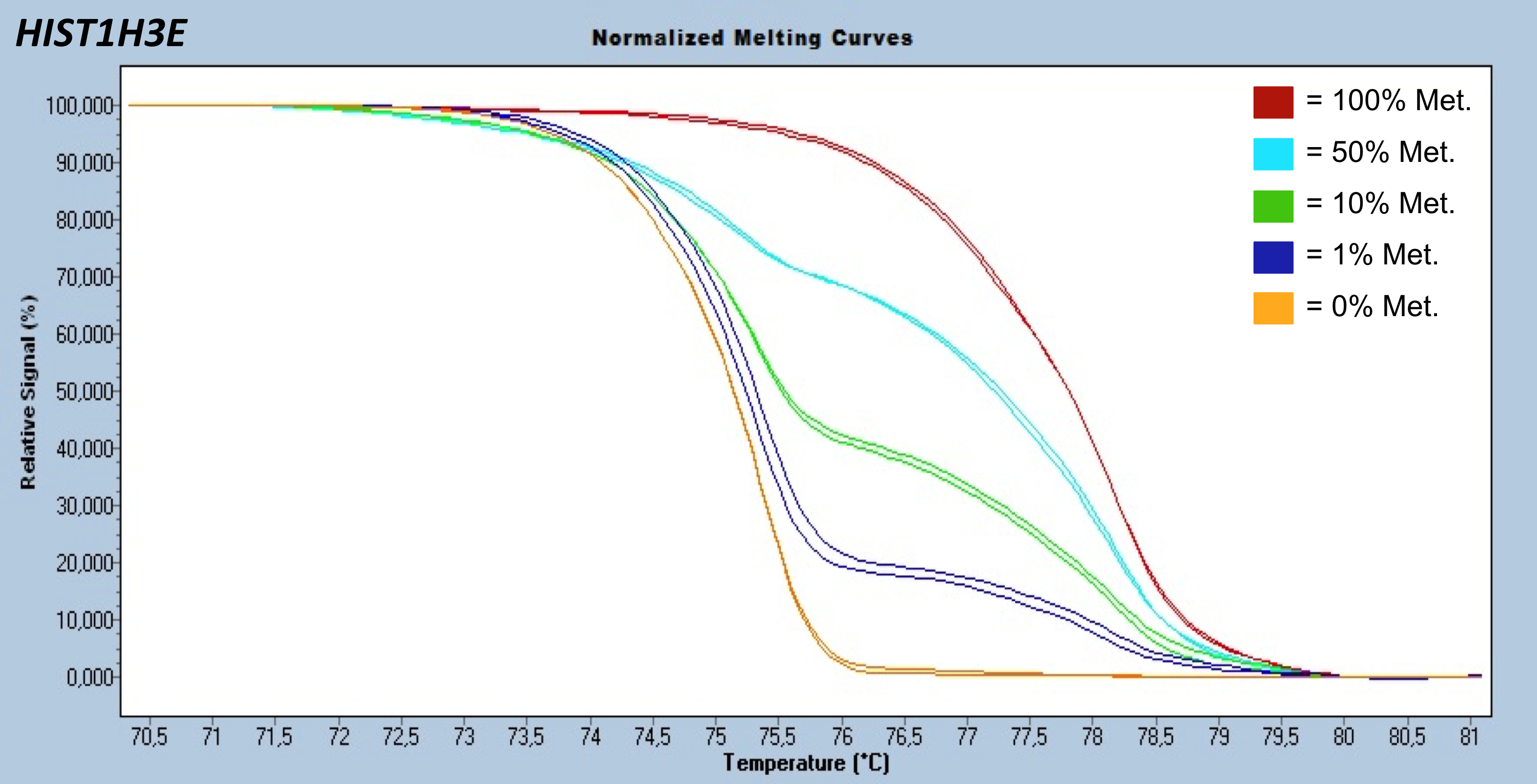

Supplement: Supplementary Information [file srep35807-s1.doc]
